# Representation of Perceptual Color Space in Macaque Posterior Inferior Temporal Cortex (the V4 Complex)

**DOI:** 10.1523/ENEURO.0039-16.2016

**Published:** 2016-08-29

**Authors:** Kaitlin S. Bohon, Katherine L. Hermann, Thorsten Hansen, Bevil R. Conway

**Affiliations:** 1 Program in Neuroscience, Wellesley College, Wellesley, Massachusetts 02481; 2 Department of Brain and Cognitive Sciences, Massachusetts Institute of Technology, Cambridge, Massachusetts 02139; 3 Department of Psychology, Justus Liebig University Giessen, 35396 Giessen, Germany

**Keywords:** color, macaque, monkey, neurophysiology, v4, vision

## Abstract

The lateral geniculate nucleus is thought to represent color using two populations of cone-opponent neurons [L vs M; S vs (L + M)], which establish the cardinal directions in color space (reddish vs cyan; lavender vs lime). How is this representation transformed to bring about color perception? Prior work implicates populations of glob cells in posterior inferior temporal cortex (PIT; the V4 complex), but the correspondence between the neural representation of color in PIT/V4 complex and the organization of perceptual color space is unclear. We compared color-tuning data for populations of glob cells and interglob cells to predictions obtained using models that varied in the color-tuning narrowness of the cells, and the color preference distribution across the populations. Glob cells were best accounted for by simulated neurons that have nonlinear (narrow) tuning and, as a population, represent a color space designed to be perceptually uniform (CIELUV). Multidimensional scaling and representational similarity analyses showed that the color space representations in both glob and interglob populations were correlated with the organization of CIELUV space, but glob cells showed a stronger correlation. Hue could be classified invariant to luminance with high accuracy given glob responses and above-chance accuracy given interglob responses. Luminance could be read out invariant to changes in hue in both populations, but interglob cells tended to prefer stimuli having luminance contrast, regardless of hue, whereas glob cells typically retained hue tuning as luminance contrast was modulated. The combined luminance/hue sensitivity of glob cells is predicted for neurons that can distinguish two colors of the same hue at different luminance levels (orange/brown).

## Significance Statement

This article provides the first quantitative test of the correspondence between the neural representation of color in posterior inferior temporal cortex (PIT; the V4 complex) and the organization of perceptual color space. fMRI-guided micoelectrode recording was used to target two subpopulations of neurons within the PIT/V4 complex, globs and interglobs. The results suggest the following: (1) glob cells have narrow color tuning, and as a population have a uniform representation of color space with a bias for warm colors; and (2) glob cells provide a neural correlate for the psychophysical distinction between two colors that have the same hue but differ in luminance (e.g., orange/brown). The work also underscores the importance of carefully controlled stimuli in neurophysiological studies of color.

## Introduction

Colors can be organized into a uniform color space in which adjacent colors are separated to a similar degree using perceptual thresholds (Munsell, 1907; MacAdam, 1990; Commission Internationale de l′Éclairage, 2004). At the same time, some colors—the unique hues (red, green, blue, yellow, black, white)—are widely considered psychologically more important than other colors (Hurvich and Jameson, 1974). The neural basis for the uniformity of color space, on the one hand, and the specialness of certain colors, on the other hand, is unknown. These features are not a trivial consequence of the spectrum: the spectrum is continuous and linear, whereas color is categorical and color space forms a circle (purple, not in the spectrum, sits where the circle closes). The retina reduces spectral information to three numbers, represented by the activity of the three, broadly tuned, classes of cone photoreceptors (L, M, S). How are cone signals processed by the brain to bring about color perception? We take up this question by asking how color is represented in a mid-tier area halfway along the putative visual-processing hierarchy [posterior inferior temporal cortex (PIT); the V4 complex]. Color-coding cells earlier on in processing, in the retina and lateral geniculate nucleus (LGN), are disproportionately sensitive to some colors (Derrington et al., 1984; Sun et al., 2006): one set of neurons responds best to colors that modulate L/M activity (without altering S activity), appearing reddish (L+, M−) or bluish-green (M+, L−); the second set responds to colors that selectively modulate S activity, appearing lavender (S+) or lime (S−). These color biases define the physiologically important cardinal directions (MacLeod and Boynton, 1979), but their impact on color categorization, if any, is not well understood. They do not correspond to the unique hues (Webster et al., 2000).

Is color space represented anywhere in the brain, such that the proportion of cells tuned to each color in the space is equal? Is there a neural correlate for unique hues? Physiology in the retina (and LGN), along with behavioral adaptation experiments (Krauskopf et al., 1982; Eskew, 2009), and some microelectrode recording studies in V1 (Tailby et al., 2008a; Horwitz and Hass, 2012), raise the possibility that color depends on a population code, in which each color is defined by the relative activation of the two sets of cardinal-tuned neurons. Other studies (Webster and Mollon, 1991; Hansen and Gegenfurtner, 2006; Stoughton et al., 2012) suggest that color depends on multiple, independent mechanisms that together comprise a uniform space. Candidate neural substrates include cells in V2 (Moutoussis and Zeki, 2002; Xiao et al., 2003) and V4 (Zeki, 1980; Li et al, 2014), and cells in subregions of inferior temporal cortex (Komatsu et al., 1992; Conway and Tsao, 2006; Conway et al., 2007; Yasuda et al., 2010; Lafer-Sousa and Conway, 2013). One possibility is that color is encoded by a population code early in processing, which is then decoded by subsequent stages (De Valois and De Valois, 1993; Zeki and Marini, 1998; Conway, 2009; Zaidi et al., 2014).

We analyzed data from fMRI-guided microelectrode recording of millimeter-sized, color-biased globs and adjacent non-color-biased interglobs in V4/PIT (Conway et al., 2007). The use of fMRI is valuable since it provides an independent means for identifying functional subdomains. Most cells in the globs are not only color tuned but also show tuning that is tolerant to luminance modulation (Conway et al., 2007; Namima et al., 2014). Moreover, glob cells are spatially organized into chromotopic maps (Conway and Tsao, 2009), consistent with a role in representing perceptual color space (Zaidi et al., 2014). A preliminary analysis suggested that the glob cells, as a population, might show a bias for the unique hues (Stoughton and Conway, 2008). Mollon (2009) has challenged this idea, arguing that variation in stimulus saturation caused the apparent biases. Mollon (2009) suggested that the data might be accounted for by a population of linearly tuned neurons biased toward the cardinal directions (such as in the LGN). To address these issues, we compared the color tuning of the population of recorded glob and interglob cells against model predictions that capture a range of theoretical possibilities incorporating the extent to which the neural tuning reflects a linear versus nonlinear combination of cone signals (narrowness of tuning), and the extent to which the color preferences across the population uniformly represent color space. The analyses suggest that the color representation in glob cells is different from the representation in the LGN: glob cells most likely possess nonlinear narrow color tuning that, as a population, represent a perceptually uniform color space with a bias toward “warm” colors (reds/yellows) over “cool” colors (blues/greens). The analyses also underscore the importance of future work to determine neural color tuning using stimuli that fully sample a uniform color space.

## Materials and Methods

### Single-unit recording

The physiological data were collected as part of a previous report (Conway et al., 2007), and all the details of the recording are described in that report. Tungsten microelectrodes were used to target microelectrode recording to functionally defined domains identified using fMRI in the same animals. Electrodes were inserted into the brain for recording sessions that lasted several hours, and then the electrodes were removed. Anatomical MR images were obtained following many microelectrode recording sessions and confirmed the placement of the electrodes. Single-unit responses were measured in two animals trained to fixate a spot on a computer monitor using standard procedures and apparatus (BAK Electronics). Complete color-tuning responses for 300 glob cells and 181 interglob cells were used in the present analysis. By combining the information from the anatomical scans and the depth information obtained during the recordings, the locations of the recorded cells were correlated with the functional maps and categorized as residing in a glob or an interglob. The anterior boundary of area V4 was initially not obvious in fMRI mapping (Fize et al., 2003), prompting use of the term “the V4 complex” or the anatomical term “posterior inferior temporal,” as used presently; subsequent evidence shows a clear boundary between V4 and inferior temporal cortex (Lafer-Sousa and Conway, 2013).

### Visual stimuli for single-unit experiments

**Figure 1.**
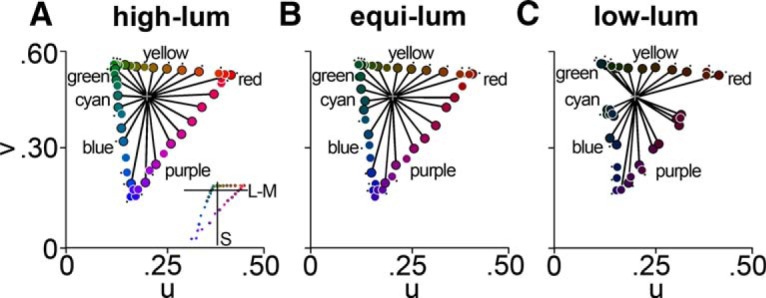
Chromaticity coordinates of color stimuli. ***A–C***, chromaticity coordinates of the stimuli in CIELUV space for the high-luminance (***A***), equiluminant (***B***), and low-luminance (***C***) stimulus sets. Cell responses to 45 hues at each luminance level were measured. Markers with black outlines denote the 21 subsampled hues. The black lines connect each of the 21 hues with the origin (gray cross). Note that the angles between each of the 21 hues are relatively uniform. Black dots denote 17 hue-matched stimuli used for decoding. Inset in ***A*** shows stimuli in MB-DKL color space.

Optimal stimulus dimensions (bar length, width, and position) were used for each cell. The shape and location were fixed for a given cell, and the color of the shape was then varied. A total of 135 colors were used, consisting of three sets of 45 colors; the colors within a set were equiluminant with each other, spanned the full color gamut of the monitor, and were as saturated as the monitor could produce (Fig. 1; Stoughton and Conway, 2008, their Table S1, CIE coordinates). The colors of one set were higher luminance (7.8 cd/m^2^) than the background; those of another set were photometrically equiluminant with the background (3.05 cd/m^2^); and those of the third set were of lower luminance than the background (0.62 cd/m^2^). All colors, including those at the lowest luminance, had discernable color to human observers. The two color sets of equal or high luminance to the adapting background were vividly colored; stimuli of the low-luminance set may be considered mesopic and could have involved rod activation. All stimuli were surrounded by an adapting background of 3.05 cd/m^2^. Luminance artifacts could cause different amplitude responses to different colors, which could be (inaccurately) interpreted as color tuning. Using stimuli at different luminance levels, and testing for luminance-invariant color tuning, provides one way of controlling for luminance artifacts arising from, for example, chromatic aberration or variability in macular pigmentation. Responses to black (0.02 cd/m^2^) and white (78.2 cd/m^2^) were also measured. The different colors were presented in pseudorandom order. Within the time period during which the three sets of colors were presented, white and black versions of the stimulus were each presented three times, so that one complete cycle consisted of 141 stimulus presentations (color set 1, 45 colors; color set 2, 45 colors; color set 3, 45 colors; white, 3; black, 3). The full stimulus set at any given luminance level did not sample the monitor gamut at regular intervals. To do so, in some analyses we subsampled the data, extracting responses to 21 hues (at each luminance level) that were more or less evenly spaced when plotted in CIELUV color space (Commission Internationale De Leclairage, 2004), which is designed to be perceptually uniform (Fig. 1, outlined points connected by spokes to the neutral point). We acknowledge that all color spaces, including CIELUV, are not entirely perceptually uniform, and the deviations from uniformity can be pronounced (Melgosa et al., 1994). To address this issue, we converted our stimuli to CIECAM02 space, a space thought to remedy some of the defects in uniformity found in CIELUV space (these defects are apparent when comparing colors across large distances in the space; Moroney et al., 2002; Luo et al., 2006). We calculated the CIECAM02 Cartesian coordinates and CIECAM02 color angle for each stimulus using the MATLAB (RRID:SCR_001622) function CIECAM02 (Computational Colour Science Toolbox; Ripamonti et al., 2013). Results using CIECAM02 were qualitatively very similar to the results obtained using the CIELUV space and did not change the conclusions (data not shown). We note that the CIECAM02 space is itself not perfect. Indeed, determining a perceptually uniform space remains a persistent challenge, both psychophysically and theoretically. Our long-term goal is to use neurophysiological data from populations of neurons shown to be involved in color to bootstrap a psychophysical definition of perceptual uniformity, and to use this information to determine the theoretical basis for the color space.

The use of monkeys to investigate the neural basis for human color perception is licensed because monkeys have very similar color detection thresholds and psychophysical mechanisms to those found in humans (Stoughton et al., 2012; Gagin et al., 2014). Responses to multiple presentations of the same stimulus were averaged together. Each stimulus was displayed for 200 ms and separated in time from the previous and subsequent stimuli by 200 ms, during which time the animal was rewarded for maintaining constant fixation.

### Estimates of stimulus saturation

The stimuli used in the original study by Conway et al. (2007) were the most saturated that the monitor could produce. The limitation of these stimuli is that there is likely considerable variability in the saturation across stimuli of different hue, confounding saturation, and hue. In an attempt to model the impact of saturation on neural responses, we estimated the saturation for each stimulus. Saturation can be defined in numerous ways, although there is no consensus; moreover, it is unlikely that the neural responses vary linearly with changes in saturation. Nonetheless, we assume linearity because the neural response to saturation has not been empirically determined. We defined saturation for each stimulus in both MB-DKL color space (a physiologically defined cone-opponent space; MacLeod and Boynton, 1979; Derrington et al, 1984) and LUV space (a perceptually defined color space). For MB-DKL saturation, we calculated the distance between the stimulus and the adapting gray point. The MB-DKL location of each stimulus was calculated with a CIE-to-MB-DKL conversion matrix from the spectra of each of the primaries of the monitor at maximum strength (Zaidi and Halevy, 1993; Hansen and Gegenfurtner, 2013). MB-DKL saturation was used to assess the hypothesis that neurophysiological data matches the activity in the LGN. For LUV saturation, we calculated the ratio of the distance between the stimulus and the adapting gray point, over the distance between the gray point and the spectrum locus through the stimulus; this definition was used to test the hypothesis that the neurophysiological data explain psychologically important colors, the unique hues.

### Preprocessing of cell responses

Every visually responsive cell that was tested was included in the analysis if responses to at least two complete stimulus cycles were obtained; in most cases, responses to at least five stimulus cycles were obtained. Most cells responded with higher firing rates compared with baseline values. A small number of cells was suppressed by the majority of stimuli at some or all luminance levels. Five glob cells and one interglob cell were on average suppressed at all luminance levels. Nine glob cells and four interglob cells were on average suppressed at one or two luminance levels. But even among these cells, none were suppressed below baseline activity by all stimuli: there was always at least one hue, at one luminance level, that elicited a response that was higher than the baseline activity. For all cells, we calculated the stimulus responses by summing spikes during a window that was optimized for the response duration of each cell, for each luminance level. The time window began with the visual latency, which was defined as >2.5 SDs above the background firing rate, and ended either when the response rate fell below the background firing rate plus 2.5 SDs, or after a period slightly shorter than the stimulus duration (reduced by one-quarter of the latency time of each cell), whichever was shorter. Capping the integration window avoided integrating over OFF responses. Across all cells, the average latency was 78 ms (SD, 28 ms). The average integration window was 146 ms (SD, 52 ms).

### Curve fitting

In order to estimate the narrowness of the response of each cell, we fit the responses with a curve. Responses to the 21 evenly spaced stimuli at each luminance level were smoothed using a boxcar filter (across one stimulus), and fit with a model tuning curve adapted from Seung and Sompolinsky (1993), according to Equation 1, as follows:

rθ=fmin+fmax*cos⁡πθ-α2w2, |θ|<wfmin,  otherwise

where $f_{min}$ is the baseline firing rate, $f_{max}$ is the maximum firing rate, w is the tuning width (full-width at half-maximum) in radians, and α is the peak tuning angle. As w increases, the cell becomes more broadly tuned; as w decreases, the cell becomes more narrowly tuned. A linear cell, such as those found in the LGN, has a w value of π radians, equivalent to 180°.

We chose to curve-fit responses to only the 21 evenly spaced in CIELUV angle stimuli in order to avoid biasing the curves to fit values closer to the monitor primaries, which were oversampled in the 45-hue set. We chose to boxcar smooth responses prior to curve fitting in order to decrease noise and improve the fit. Results obtained using all 45 stimuli and unsmoothed responses yielded similar conclusions. Results obtained using a half wave-rectified cosine exponent curve (De Valois et al., 2000) yielded similar conclusions (data not shown).

Is it possible that our sampling of colors space (21 angles ∼17° apart) was too coarse to obtain a good estimate of narrowness? To assess this, we compared the tuning width estimates with those obtained using responses to all 45 hues. If a 21-hue set is insufficient to provide an accurate estimate of tuning width, we would expect the tuning widths to be narrower when using the responses to more dense sampling of color space, especially for cells with tuning peaks located in the part of color space most densely sampled by the 45 colors. The 45-color stimulus set sampled most densely the color space around the monitor primaries. We did not find systematic differences of the tuning widths estimated with either approach (data not shown), suggesting that 21 hues sampled densely enough to accurately reflect the tuning widths of the neurons.

### Model populations

The color space represented by a population of neurons can be defined by varying the following two parameters: the narrowness of the color-tuning function for each cell (w); and the uniformity with which the population of neurons samples color space. Our goal was to determine the combination of these parameters that best describes the color representation in the globs and interglobs, and to compare these parameters to those evident in the LGN. We performed multiple iterations of a model simulation, parametrically varying the linearity and degree of uniformity in a population of model cells; we used 181 model cells on each iteration of the model (corresponding to the number of cells recorded in the interglobs). To compare the model to the glob population, we randomly sampled 181 units from the total 300 glob cells recorded. This subsampling was performed in order to equate the number of cells between the glob and interglob populations to allow a direct comparison of the best-fitting models achieved for the two populations of neurons.

To simulate an entirely uniform population of linear glob cells, each model cell was assigned a tuning width of 180, and a random peak tuning angle drawn from a uniform distribution of integers between 1 and 360. A tuning function was generated using Equation 1. The baseline firing rate and maximum firing rate were not varied, as all responses (both model and real cells) were normalized to facilitate comparison between recorded and model cells; for the simulated neurons, the minimum firing rate was set to 0 and the maximum firing rate was set to 1. To account for differences in saturation among the stimuli, the response of the model cell to each stimulus was multiplied prior to normalization by the saturation of that stimulus, as described above. A stimulus with higher saturation would more greatly affect the tuning curve of the model cell than a stimulus with lower saturation. The 181 model cells were then rank-ordered by the angle to which they showed peak response. The neural populations were also rank ordered by the LUV angle at which they maximally fired. *R* ^2^ values were then computed between the tuning function of each model cell (defined by both tuning width and peak tuning angle) and its corresponding (rank-ordered) recorded neuron (raw responses to all 45 hues). This procedure was performed 1000 times. The success of the match between the population of recorded cells and the model simulation was defined as the median *R* ^2^ value across all 181,000 comparisons. We determined the *R* ^2^ values for simulations in which the narrowness varied from 84 to 360 CIELUV degrees. The model responses were compared with unsmoothed responses obtained to all 45 stimuli of a given luminance level.

We also determined *R* ^2^ values for simulations in which the uniformity of color space varied. We tested the following two hypotheses: first, that the population reflected the distortions of the color space manifest in the retina and LGN; and, second, that the population reflected the distortions of color space predicted by the purported privilege of the unique hues. To simulate a population of LGN cells, each model cell had a randomly assigned α drawn from a distribution of values within 5° of the cardinal angles in LUV space (353°, 100°, 173°, 280°). To simulate a population of unique hue-biased cells, each model cell had a randomly assigned α drawn from a distribution of values within 5° of the unique hue angles (14°, 70°, 139°, and 238°). The degree of nonuniformity within each model simulation was then systematically varied by adjusting the fraction of the model cells that were defined as nonuniform (LGN or unique hue) versus uniform.

We summarize the conclusions of the model simulations in heat maps of the median *R* ^2^ values across the 181,000 comparisons (1000 iterations × 181 recorded-model cell pairs) at each narrowness–uniformity pairing. The darker the cell of the heat map, the better overall correlation there was between the model population and the real population. Black/white boxes indicate the best-matching simulated population, and numbers report the median *R* ^2^ for the best-matching simulated population.

### Receiver operating characteristic analysis

To test whether cells in the glob and interglob populations discriminate stimuli based on luminance, we performed a receiver operating characteristic (ROC) analysis (Britten et al., 1992; Mayo and Sommer, 2013). We *z*-scored the raw mean firing rates of each cell to the full stimulus set (45 hues at each of three luminances). For each cell, for each pair of luminance categories (equiluminant/low-luminance, high-luminance/equiluminant), we used the perfcurve function in Matlab R2013b to compute an area under the curve (AUC) of the ROC of the cell. An AUC of 0.5 would indicate chance discrimination between the two luminance categories. An AUC < 0.5 would indicate a preference for the first luminance category. An AUC > 0.5 would indicate a preference for the second luminance category. To determine whether the AUC of a cell was significantly different than chance, we performed a permutation test in which, for each of 2000 iterations, we performed the ROC procedure but with randomly shuffled luminance category labels. This yielded a null distribution for which we computed 95% confidence intervals (CIs); if the AUC of a cell fell outside these bounds, it was deemed significant at *p* < 0.05.

### Analysis of peak shifting as a function of luminance

To determine the effect of stimulus luminance on the glob and interglob hue preferences, we quantified the change in color-tuning preferences across luminance levels. We sought to test whether or not cells maintained the same hue-tuning preference across luminance levels; for example, does a cell that responds best to purple at equiluminance also prefer purple at high-luminance? This test sheds light on the possible involvement of rods. Although the adapting state maintained by the surrounding neutral gray was likely photopic (3.05 cd/m^2^), the stimuli of the low-luminance set had relatively low luminance, raising the possibility that they activate rods. Moreover, some prior work would also suggest that the stimuli of the other luminance sets might also involve rods. If rods were implicated in driving the neural responses, one might expect systematic shifts in color tuning as the luminance is changed (Shapiro et al, 1996). Our results do not provide conclusive evidence for such shifts (see Fig. 9).

To assess the extent to which neurons of each population (glob and interglob) shifted their peak hue tuning, we compared the correlation of the peak tuning determined at different luminance levels. We calculated the Pearson’s correlation coefficient (*r*) between the peak determined using stimuli at one luminance level and the peak determined using stimuli at a different luminance level following 200 bootstraps of the responses of half of each population. We performed a *t* test on the glob and interglob distributions of Fisher’s *z*-transform-corrected *r* values. In order to calculate 95% CIs on these *p* values, we performed the 200 bootstraps 1000 times, and calculated the CIs using the percentile method. The reported *p* value for each comparison is the median of the 1000 *p* value distribution.

In order to test for systematic differences in peak shifting across luminance levels between cells tuned to different hues, we calculated peak shifting within groups of neurons defined by their color preferences assessed using the equiluminant stimulus set. We categorized the cells into eight color categories, each spanning 45° in color space. We used a Mann–Whitney–Wilcoxon *U* test to determine whether the tuning of the population in each category shifted when tuning was assessed at different luminance levels. The 95% confidence intervals on this *p* value were obtained by doing 2000 bootstraps, in which the *p* value was calculated using a random selection of 90% of the cells. The reported *p* values are the mean *p* value over these bootstraps. This analysis was performed for both glob cells and interglob cells, comparing tuning at equiluminance to tuning at low-luminance, as well as tuning at high-luminance to tuning at equiluminance. The Seung–Sompolinsky curve fits (Eq. 1) were used to determine the peak tuning angle at each luminance level.

### Multidimensional scaling

To view the population representations of stimuli, we applied multidimensional scaling (MDS). Each stimulus has a high-dimensional neural representation, with each dimension corresponding to the raw mean firing rate of a single neuron in response to that stimulus. MDS attempts to find a *k*-dimensional embedding of this high-dimensional space that approximately preserves its structure, where *k* is specified as an input. Given a set of *x* stimuli *S* = {*s*_1_, … *s_x_*} and a function *d* measuring the pairwise dissimilarities between them, MDS uses an iterative algorithm to find an embedding $f:\;S\to R^{k}$ for some fixed *k* such that the distances $\left|\left|f\left(s_{i}\right)-f\left(s_{j}\right)\right|\right|$ have approximately the same rank ordering as the dissimilarities *d*(*s_i_*, *s_j_*). We selected Sammon’s error (Sammon, 1969) as the error function to minimize, and defined the dissimilarity between two stimuli *s_i_* and *s_j_* in terms of their neural representations $\hat{s_{i}}$ and $\hat{s_{j}}$: $d\left(s_{i},s_{j}\right)=1-\rho (\hat{s_{i}},\;\hat{s_{j}})$, where ρ is Pearson’s correlation coefficient. Stimuli are considered dissimilar to the extent that the population responses they evoke are uncorrelated.

We performed two separate MDS analyses on the glob and interglob population datasets. The aim of the first analysis was to view how the populations represent the full stimulus set (45 hues at each of three luminances). We *z*-score normalized the raw response of each neuron to the stimulus set, and applied MDS for a range of *k-*values. In the second analysis, we examined separately the neural representations of each stimulus luminance class (stimuli at lower luminance than the background, equiluminant with the background, and at higher luminance than the background) in order to view the neural representations of hue within each luminance class. Here, we *z*-score normalized the responses of each neuron within each luminance set (45 hues each) before performing MDS for *k* = 2.

### Representational similarity analysis

We used representational similarity analysis (RSA; Kriegeskorte et al., 2008) to compare the neural representations of hue by the two populations to CIELUV color space hue angle. We divided the data into three luminance sets (45 hues each) as above, and *z*-score normalized the response of each neuron within a set. For each set, we created a neural representational dissimilarity matrix (RDM) in which, as in the MDS analyses, an entry contained the neural correlation dissimilarity between two stimuli. We created a second RDM containing the CIELUV hue angle distance between stimuli. For each luminance class, we then determined the Pearson's *r* value between the neural and CIELUV RDMs, yielding a measure of similarity between neural and color space representations of hue (Cichy et al., 2014). To test whether these correlations were significant, we performed a Student’s *t* test, two-tailed on the Fisher’s *z*-transforms of *r*. To determine whether there was a significant difference between pairs of correlation coefficients for the glob versus interglob populations, we performed paired *t* tests, two tailed to *z*-transforms of the *r* values.

### Model LGN populations

To test whether or not the MDS and RSA results for the glob and interglob cells were different than those we could expect earlier in the visual system, we performed these analyses on a population of model parvocellular LGN cells. The population of 300 model LGN cells was generated using the narrowness-uniformity model (see Fig. 5). The model LGN cells were linearly tuned (tuning width of 180) and biased for the cardinal axes (28% uniform, 72% cardinal), matching values found in previous studies of the LGN (Derrington et al., 1984; De Valois et al., 2000). The model does not account for luminance, so we analyzed the responses to the luminance levels separately. Limitations of the model also precluded an MDS analysis of the full dataset for comparison with the MDS analysis performed on the glob and interglob cells (see Fig. 10*A*,*B*). Additionally, because the model LGN cells had identical responses to a small number of stimuli, these stimuli were removed from analysis. MDS was run on responses to 41 low-luminance stimuli, 43 equiluminant stimuli, and 45 high-luminance stimuli.

### Decoding hue and luminance information from the population responses

Guided by the results of the MDS analyses and RSAs, which suggested that both populations represent both hue and luminance information, we sought to determine (in the population responses) whether hue information was preserved across changes in luminance, and whether luminance information was preserved across changes in hue. We determined accuracies in classifying (1) hue information invariant to changes in luminance, and (2) luminance information invariant to changes in hue, using a linear support vector machine (SVM; MATLAB_R2013b svmtrain, least-squares method), which attempts to find a hyperplane with maximum margins separating the high-dimensional points (neural responses to stimuli) belonging to two training classes. For these analyses, we used responses to a hue-matched subset of the stimuli (described in the next paragraph), and, prior to applying decoding, *z*-score normalized the responses of each neuron within the hue-matched stimulus subset.

For our decoding analyses, it was important that we used sets of stimuli that had closely corresponding hue angles across the luminance levels. We identified a triplet of hue-matched stimuli as one in which the three hues at the three luminance levels differed no more than 3° in CIELUV angle. Seventeen hue-matched triplets (51 stimuli) were identified (Fig. 1, colors identified by a small dot).

In order to test the decoding of hue invariant to changes in luminance, we tested whether a linear SVM could generalize hue information from two luminance classes to a third luminance class. For each possible pair of the 17 hues in the hue-matched stimulus subset, we trained the classifier to distinguish between the pair of hues, *h*_1_ and *h*_2_, given the population response to these hues at two luminances, and tested whether the classifier assigned the correct labels to *h*_1_ and *h*_2_, given the population response to the test luminance (e.g., high luminance). We performed decoding for three generalization problems, generalizing to low-luminance, equiluminant, and high-luminance stimuli. We present the mean pairwise decoding accuracies for each of these problems separately.

In order to test the decoding of luminance invariant to changes in hue, we tested whether the classifier could generalize luminance information across changes in hue. We performed three decoding problems: we trained and tested a classifier’s ability to distinguish among the (1) low-luminance and equiluminant stimuli, (2) equiluminant and high-luminance stimuli, and (3) low-luminance and high-luminance stimuli. For each classification problem, we trained the classifier on 15 of 17 stimulus hues, and tested on the held-out pair. We performed a decoding run for each possible pair of test hues. We present the mean decoding accuracy across runs for each of these classification problems (see Fig. 12).

In both decoding analyses, to account for a mismatch in population size between the globs (*N* = 300) and interglobs (*N* = 181), we performed subsampling. For each of 200 subsampling runs, we drew a random subset of 181 glob neurons, and performed the full decoding procedure to obtain decoding accuracies for this population.

To test whether decoding accuracies for each classification problem were significantly above chance, we performed a permutation test in which we repeated the full decoding procedure 200 times with randomly shuffled labels, yielding a null distribution of decoding accuracies. We counted as significant decoding accuracies lying above all null points, which enabled us to bound *p* at 0.005 (1/200). For the glob population, this procedure was repeated for each subsampling run (all subsampling runs achieved accuracy at *p* < 0.005). To test whether decoding accuracies were significantly different between the glob and interglob populations, for each classification problem, we obtained a *p* value by taking the average of 200 *p* values derived by comparing the results for the interglob population and the results for one subsampling run of the glob population using a two-tailed McNemar’s exact test.

### Comparing narrowness of tuning with estimates obtained in prior work

In order to compare the narrowness of the tuning of cells in the glob and interglob populations with values reported by Namima et al. (2014) for cells in V4, PIT, and anterior inferior temporal cortex (AIT) populations, we followed a similar method to compute a narrowness measure. We considered the responses of each cell to the low-luminance and high-luminance stimuli in the hue-matched subset, to match the high- and low-luminance stimulus sets used in the analysis by Namima et al. (2014). For each cell, for each luminance set, we calculated a selectivity index = 1 − (minimum response)/(maximum response), where responses are the raw mean firing rates of the cell in response to the luminance set. If a cell had a selectivity index >0.6 for either luminance set (all cells in the glob and interglob populations met this criterion), we next computed the sparseness index of that cell (Rolls and Tovee, 1995; Vinje and Gallant, 2000) using Equation 2:

$$sparseness\;index=\;\left[1-\frac{(\overset{n}{\underset{i=1}{\sum }}\frac{r_{i}}{n}{)}^{2}}{\left(\sum_{i=1}^{n}\frac{r_{i}^{2}}{n}\right)}\right]/\left(1-\frac{1}{n}\right)$$

where *r_i_* is the response of the cell to the *i*th stimulus and *n* is the number of stimuli in the luminance set. A sparseness index of 1 indicates the cell is sharply selective, whereas a low score indicates the cell responds similarly to all stimuli. Consistent with Namima et al. (2014), we then labeled cells with sparseness indices >0.3 for either set as “sharply selective,” and those with sparseness indices ≤0.3 to both sets as “broadly selective.” We compared the proportions of sharply tuned and broadly tuned cells in the two populations to values reported by Namima et al (2014, their Fig. 5).

### Proportion of warm- and cool-tuned cells

In order to determine whether the glob or interglob populations were biased for warm colors over cool colors, as suggested in the population-tuning distribution for the glob cells (see Fig. 3), we performed a permutation test. We defined warm hues as the CIELUV hue angles lying between Munsell RP and Y (pink, red, orange, and yellow), and cool hues as those between Munsell G and PM (green, cyan, blue, and violet). On each of 2000 permutations, we randomly assigned a population of cells (*n* = 300 for globs, *n* = 181 for interglobs) 1 of the 45 hue angles per luminance level. We then calculated the ratio of cells tuned to warm colors to those tuned to cool colors. We then used this distribution to calculate *p* values. For 2000 permuations each, using a random selection of 90% of the real glob or interglob populations, we calculated the warm-tuned-to-cool-tuned cell ratio. We then determined a *p* value by counting the number of permutation populations with a higher warm-tuned-to-cool-tuned cell ratio than the bootstrap population. The 95% CIs on the *p* value were also calculated from the bootstrap distribution using the percentile method. This analysis was performed separately for both populations (globs and interglobs), and each luminance level.

## Results

The neurophysiological data were obtained from the original study by Conway et al. (2007). The stimuli used to characterize the color responses were defined using the CIELUV chromaticity diagram (Komatsu et al., 1992; Conway et al., 2007), which organizes colors in a more or less perceptually uniform manner (Fig. 1). The full stimulus set comprised 45 colors at three luminance levels (all the colors within a set were equiluminant with each other; one set was higher luminance than the adapting background; one set was lower luminance; and one set was equiluminant with the background). Of these 45 colors, 21 hues were selected to be at relatively equal angles in CIELUV space (Fig. 1, points outlined in black); responses to these stimuli were used to quantify the neural color tuning. In other analyses, we analyzed responses to 17 hue-matched (within 3°) stimulus triplets (Fig. 1, dotted colors); responses to these hue-matched stimuli were used in various decoding analyses, which are described below.

**Figure 2.**
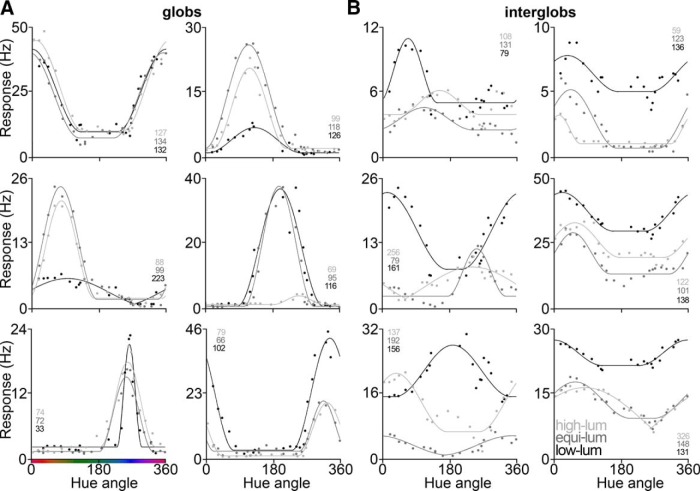
Color tuning of representative glob and interglob cells. ***A***, ***B***, Responses to the 21 subsampled hues, smoothed with a boxcar kernel of 1 hue, at each luminance level from six glob cells (***A***) and six interglob cells (***B***). Responses were measured using a bar stimulus optimized for each cell. Points show spike/second (Hz) firing rate response to each stimulus’s LUV hue angle. Lines show the Seung–Sompolinsky curve fit (light gray, high-luminance set; dark gray, equiluminant set; black, low-luminance set). Numbers denote the narrowness (tuning width in CIELUV degrees) for each example cell.

Figure 2 shows tuning curves obtained for representative cells in the globs (Fig. 2*A*) and interglobs (Fig. 2*B*). Glob cells typically showed narrower color-tuning curves than interglob cells, and color preferences that were retained across different luminance levels. The median tuning width (see Eq. 1) among glob cells was narrower than that of interglobs for all luminance levels (glob cells: 104°, 84°, and 91°, respectively, for high-luminance, equiluminant, and low-luminance; interglob cells: 120°, 125°, and 121°). For the combined population of glob and interglob cells across all luminance levels, the median narrowness was 100. The median goodness of fit of the tuning-curve fits for glob cells and interglob cells were 0.86 and 0.62. The correlation of peak tuning preferences across luminance levels was higher for globs (0.90 equiluminant/low-luminance; 0.91 high-luminance/equiluminant) than interglobs (0.80 equiluminant/low-luminance; 0.84 high-luminance/equiluminant). See Figures 8 and 9 for more in-depth population analyses of narrowness differences between the two populations and tuning differences across luminance levels.

**Figure 3.**
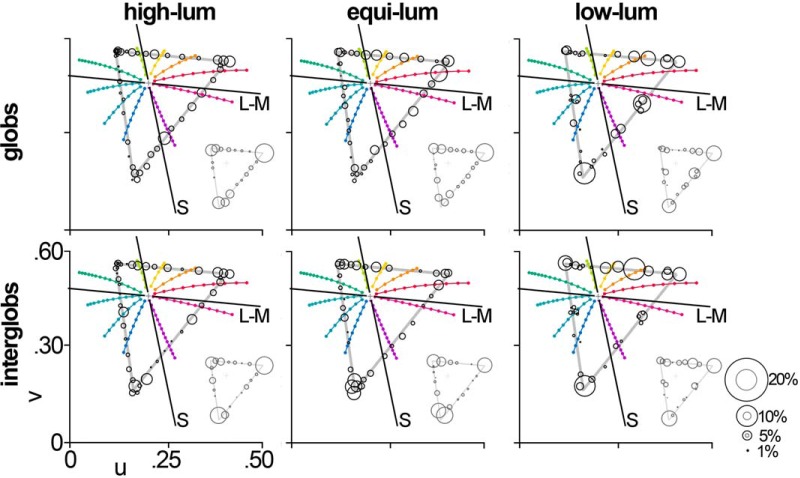
Peak color tuning distributions for the glob and interglob populations. The peak color tuning of each cell at each luminance level was defined as the angle of the stimulus to which each cell maximally fired. The number of cells tuned to each hue was counted. The size of the marker at each hue denotes the number of cells tuned to each hue. This analysis was performed separately for the glob (top row) and interglob populations (bottom row), for all 45 stimuli (main plots) and then binned into the 21 evenly spaced hues (insets). In all panels, black axes labeled L-M and S denote the cardinal axes, and colored markers show the nine Munsell primary and intermediate hue coordinates.

Figure 3 shows the number of cells tuned to each of the 45 colors at each luminance level, for globs and interglobs. For each cell, the color tuning was defined as the color corresponding to the stimulus that elicited the peak firing rate. We applied no smoothing of the firing rates across colors, to avoid inflating the extent to which our model predictions, described below (see Fig. 5), correspond to the neural data. (As expected, comparisons of smoothed neural data and the model predictions produced higher *R* ^2^ values; data not shown.) The insets in Figure 3 show the distribution of color-tuned cells, binned into categories defined by the 21 equally spaced hues. The plots show lines demarking the cardinal axes (L-M, and S), along with the Munsell principal and intermediate hues (Munsell, 1907). The red, yellow, green, and blue lines are the Munsell coordinates corresponding to the unique hues, although we acknowledge that there is considerable variability within the population with regard to the precise location of the unique hues. Both the glob and interglob populations included neurons tuned to every stimulus we used. Both glob and interglob cells showed a clear over-representation of some colors (red, green, blue, and possibly purple). The pattern of over-representation was evident at all three luminance levels tested, but more clearly consistent across luminance levels for the glob population.

To test whether the populations are best described by a distribution biased toward the cardinal directions, by the unique hues, or, alternatively, by a uniform distribution, we compared the neurophysiological results to model predictions. The simulated neural populations corresponded to a range of theoretical possibilities by varying the uniformity with which the population represents color space (100% uniform vs a bias toward either the cardinal colors or the unique hues), and the linearity of the color tuning of the cells (100% linear, meaning a tuning curve in the shape of a sine wave, vs highly nonlinear, meaning a tuning curve narrow than a sine wave). Cells with maximally nonlinear tuning would respond to a single color only. Importantly, the model incorporates information about the saturation of the stimuli to account for the differences in saturation among the stimuli used to test the neural responses. Plotted in CIELUV space (Fig. 1) or cone-opponent MB-DKL space (Fig. 1*A*, inset), the stimuli track a triangle defined by the three monitor primaries. Consider a cell that shows maximal responses to color X when tested with a stimulus set comprising colors of equal saturation (a circular set). “X” would constitute the true color tuning of the neuron. But now consider the response of the same cell when tested with the triangular set: the cell could show peak firing to an adjacent color of higher saturation, especially if the tuning of the cell is relatively broad (Mollon, 2009). We sought to test whether the biases in the population reflect those predicted for a population of linear cells that represent color space uniformly, against the two other predictions: a population of linear cells that over-represents the cardinal directions, and a population of nonlinear cells that over-represents the unique hues.

**Figure 4.**
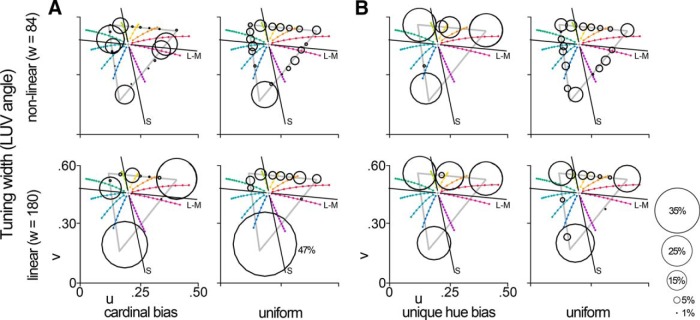
Peak color tuning distributions predicted by model populations. Like recorded cells, the peak color tuning of each model cell at each luminance level was defined as the stimulus to which each cell had the largest response. Model cell responses were the product of the Seung–Somplinsky tuning curve and the saturation of each stimulus. ***A***, ***B***, Conventions are as in Figure 3, for model populations bias for the cardinal axes (***A***) and the unique hues (***B***). Shown here for the 21 evenly spaced equiluminant hues. Cardinal distribution is 22% uniform and 78% cardinal (our closest approximation of the LGN); unique hue distribution is 100% unique hue biased; uniform distributions are 100% uniform.

We determined how the different model populations would respond to the 21 evenly spaced colors of the equiluminant triangular stimulus set (Fig. 4). The lower left panel of Figure 4*A* shows the predicted color-tuning distribution for a population of linear neurons that as a population are biased for the cardinal axes, simulating the known properties of LGN cells. The population shows two dominant peaks, which correspond to the colors of highest cone contrast and highest saturation (within the stimulus set, these colors are closest to the red and blue primaries of the monitor). The lower right panel in Figure 4*A* shows the distribution for a population of linear neurons that sample color space uniformly; again, the population distribution is weighted toward the color of maximum saturation (blue), although there are subsidiary peaks for intermediate colors. The top panels in Figure 4*A* show the corresponding distributions for nonlinearly tuned neurons. The predicted population responses are less distorted by differences in saturation: the top left panel in in Figure 4*A* shows four peaks, corresponding to the poles of the cardinal axes; the top right panel in Figure 4*A* shows peaks sampling the entire gamut. Figure 4*B* shows predictions for model populations that are biased toward the unique hues; the model predictions for populations of uniformly tuned neurons shown in Figure 4*B* are different from the predictions for populations of uniformly tuned neurons shown in Figure 4*A*: a metric of saturation defined in psychophysical color space (CIELUV) was used in Figure 4*B*, while a metric of saturation defined in cone-opponent coordinates was used in Figure 4*A* (see Materials and Methods).

### Comparison of recorded and model populations

**Figure 5.**
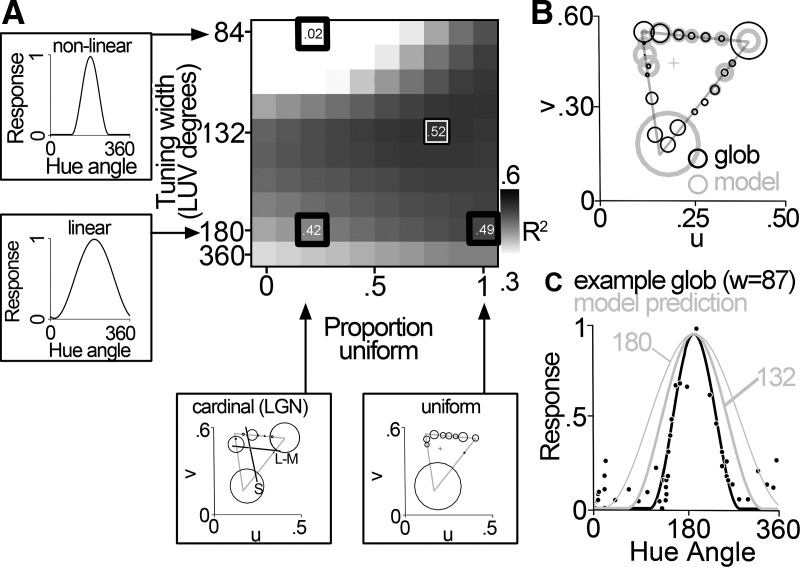
Model of color-tuning properties. We generated model populations varying in peak tuning distributions and narrowness. Each simulated cell was assigned a peak tuning angle that was drawn either from a distribution centered on the cardinal angles, or a uniform distribution. ***A***, The proportion of simulated peaks drawn from each distribution was systematically varied from 100% cardinal to 100% uniform (bubble plots). ***A***, All cells in each iteration were assigned the same narrowness, which varied from more broad than linear (tuning width of 360) to highly nonlinear (tuning width of 84; tuning curves) and then scaled by the saturation of each stimulus. Each square of the heat map represents one combination of narrowness and proportion uniform peaks. Each model population was compared with the recorded glob or interglob populations. The darker the square of the heat map, the better overall correlation between the model population and the real population. The best-matching simulated population is indicated with a black/white box. The heat map shown here compares the responses of the model population to the responses of the glob population to the equiluminant stimuli. Thick black boxes correspond to the example peak distributions and tuning curves to the left and below the heat map; the black box in the lower left shows the predicted best-matching population for the LGN. ***B***, ***C***, The best-matching model population was similar to the glob cells in both tuning peaks (***B***; conventions are as in Fig. 3) and tuning width (***C***; black line average glob tuning curve, black points show representative glob cell, thick gray line shows tuning function of the best model fit, thin gray line shows tuning function of a model cell with linear narrowness.).

Figure 5 quantifies the relationship between the measured population responses and those predicted by one set of models. The tuning curves for each cell in the model were drawn either from 10°-wide distributions centered on the cardinal hues or from bins that uniformly sampled color space. To define the population of cells, the proportion of cells with peaks drawn from the cardinal categories was systematically varied, from all cells drawn from drawn from the cardinal categories (a value of 0 on the *x*-axis) to all cells drawn from the uniform sampling (a value of 1 on the *x*-axis). All cells in each iteration were assigned the same narrowness, which varied from broader than linear to highly nonlinear (tuning curves were modeled using Eq. 1; we varied the tuning-width parameter to achieve nonlinear tuning). The blackness in the heat map in Figure 5*A* corresponds to the *R* ^2^ value comparing the model prediction to the neural glob data obtained using the equiluminant stimulus set. The model that captures the pattern in the LGN (linear cells tuned to the cardinal axes) yielded a relatively low *R* ^2^ value (Fig. 5*A*, outlined black box in the lower left). We can therefore rule out the first hypothesis that the population is best described by linearly tuned cells that are biased for the cardinal directions. Could the neural data be well described instead by a population of linear neurons regardless of the population distribution? No: the *R* ^2^ values obtained for models capturing nonlinear tuning tended to be greater (the rows in the heat map get darker from bottom to top up until the best-fitting width of 132). Across the columns in the heat map, the *R* ^2^ values are highest for the models that capture an almost completely uniform representation of color space (far right columns in the heat map). Of these, the optimal model is the one that consists of cells with nonlinear color tuning (*R* ^2^ = 0.52; narrowness = 132; Fig. 5*B*). That the best model fit consists of nonlinear neurons is reflected in the tuning curves of the recorded data (Fig. 2). Figure 5*C* shows the tuning curve and raw data for an example glob cell with median narrowness (black line), along with a model tuning curve with a tuning width of 132 (and for comparison, the linear curve, tuning width of 180; see Fig. 8). These results undermine the hypothesis that the population comprises a uniform distribution of linear neurons that manifests as a population with biases for those colors of highest saturation.

**Figure 6.**
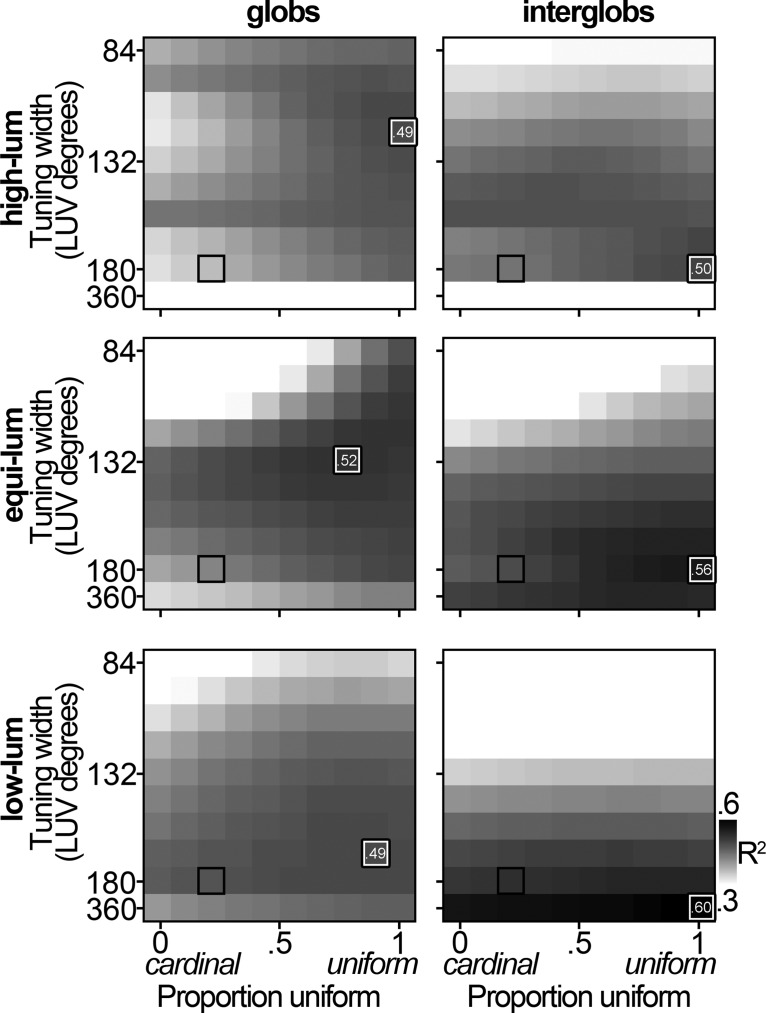
Comparison with model populations: cardinal. Heat maps in the convention described in Figure 5 show the median *R* ^2^ value across the 1000 iterations of the model and 181 recorded-model cell pairs for the glob (left) and interglob (right) populations. Darker shading denotes better fits. White/black outline denotes the parameter combination with the highest median *R* ^2^ value for that luminance level. Top row, High-luminance stimulus set; middle row, equiluminant stimulus set; bottom row, low-luminance stimulus set. Black box to lower left denotes the best expected match for LGN cells.

The best model was similar regardless of the luminance of the stimulus set used to collect the neural data (Fig. 6, left panels): for each stimulus set, the best model was one comprised of nonlinear neurons that represent color space in a uniform fashion. Glob cells were best matched by cells with nonlinear tuning for the high-luminance and equiluminant stimulus sets (tuning width of 120 for the data obtained using the high-luminance sets, 132 for the equiluminant set, and 168 for the low-luminance set). Interglob cells were best fit by a population of broadly tuned neurons with a uniform tuning distribution (Fig. 6, right panels).

**Figure 7.**
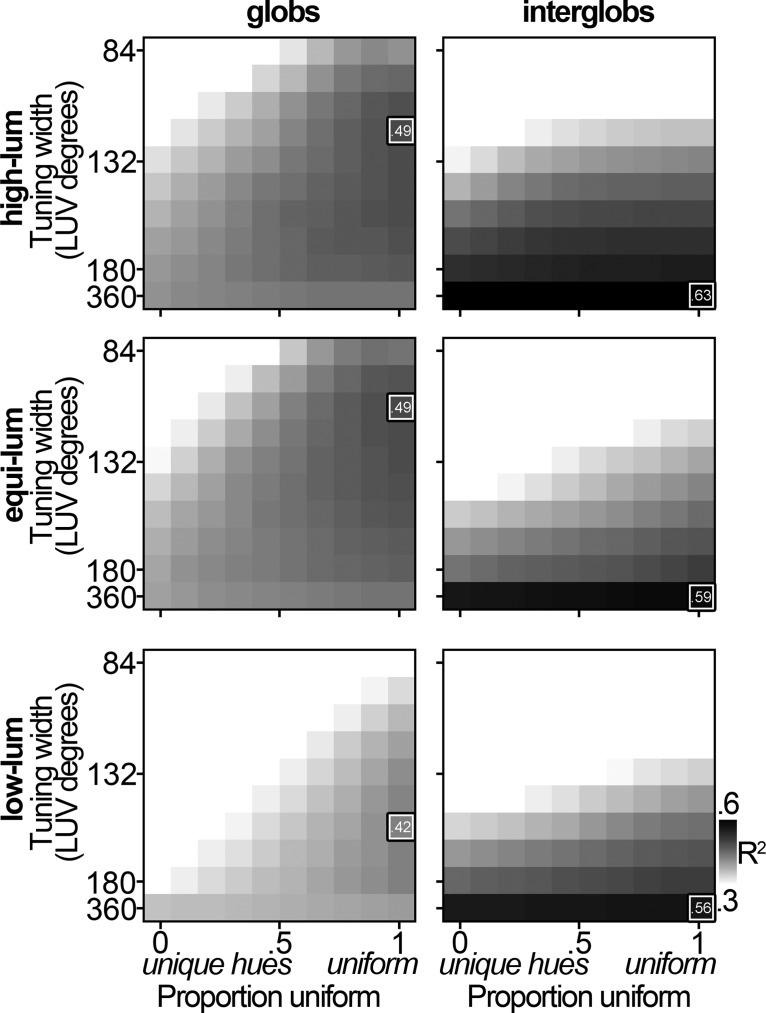
Comparison with model populations: unique hues. Heat maps with conventions as in Figure 5 for glob (left) and interglob (right) populations. Peak distributions ranged from 100% unique hues to 100% uniform. Top row, High-luminance stimulus set; middle row, equiluminant stimulus set; bottom row, low-luminance stimulus set.

The preceding results show that the population of PIT neurons cannot be well described by a model comprising neurons, such as those in the LGN, that are biased for the cardinal directions. But can the population be better explained by a model biased for the unique hues? Figure 7 shows the heat maps comparing the neural data with simulated populations that vary in the extent to which the neurons are biased for the unique hues. The models that account for the most variance among the glob cells are those that comprise nonlinear neurons with a uniform representation of color space. The interglobs are best described by the model populations consisting of broadly tuned neurons. Neurons with broader tuning functions are more sensitive to variation in stimulus saturation, which would lead to peaks in the population distribution for colors of highest saturation (Fig. 4, bottom rows, peaks at the apices of the triangles). The conclusions from Figure 7 are no different if saturation is defined in cone-opponent space (data not shown).

### Narrowness in the recorded populations

**Figure 8.**
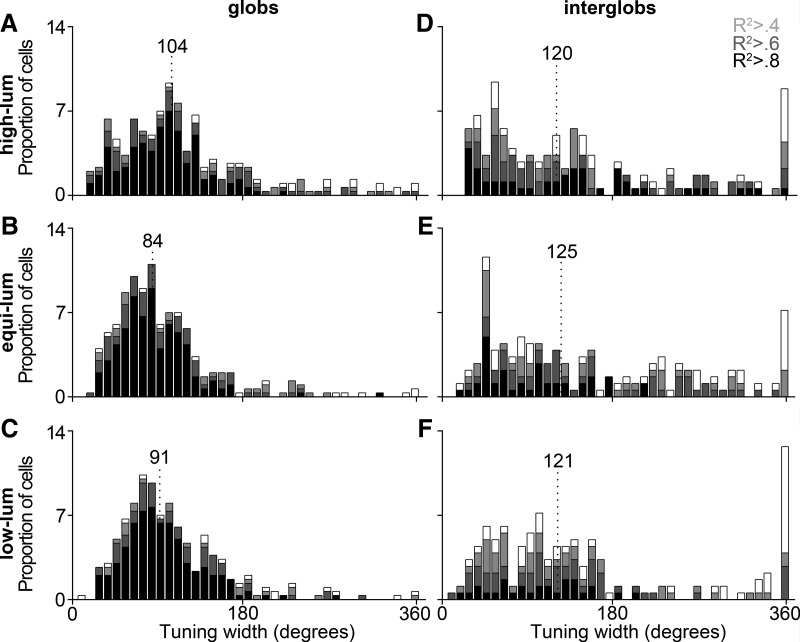
Tuning width distributions. The responses of each cell were fit with a Seung–Sompolinsky curve equation (see Materials and Methods). As the narrowness of the tuning of the cell increases, the tuning width parameter decreases. Glob cells (***A-C***) had lower tuning width values on average than interglob cells (***D-F***) for all luminance levels. Top row, Histogram for high-luminance stimuli; middle row, equiluminant stimuli; bottom row, low-luminance stimuli. Dotted line marks the median tuning width. Shading indicates the goodness of fit of the sine exponent curve: white, all cells; light gray, cells with curve fits of *R* ^2^ > 0.4; dark gray, *R* ^2^ > 0.6; black, *R* ^2^ > 0.8.

In order to relate the parameters predicted by the model to those found in the recorded cells, we quantified the tuning properties of the glob and interglob cells. First, we fit each cell with the Seung–Somplinsky curve fit (Eq. 1; Figs. 2, 5*C*, curve fits; we used the same equation, multiplied by the saturation of each stimulus, to simulate cell responses in the model). Glob and interglob cells showed a variety of tuning curve widths, but, in general, glob cells showed narrower tuning compared with interglob cells (Fig. 8). The majority of poor fits for the interglobs were cells with very broad (often almost flat) color-tuning curves (Fig. 2*B*, bottom left, response to equiluminant stimuli for the example cell).

### Effect of stimulus luminance on recorded cell responses

**Figure 9.**
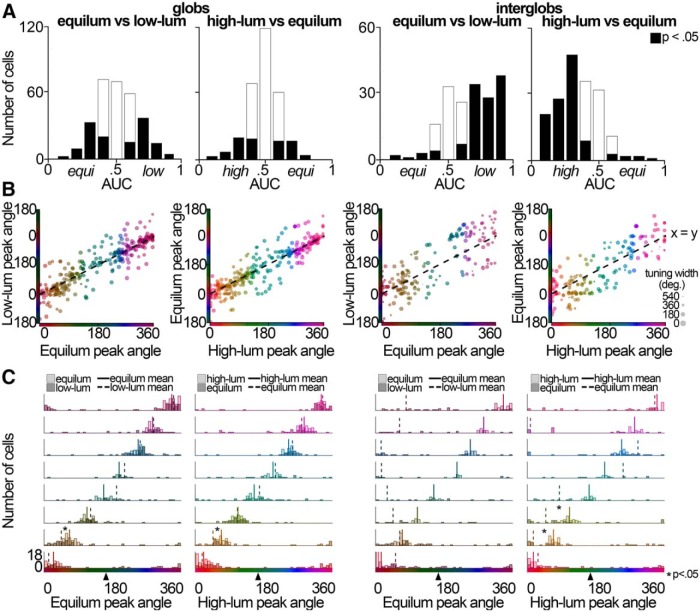
Effect of stimulus luminance on tuning. ***A***, Luminance sensitivity of cells in the glob (left) and interglob (right) populations assessed by ROC analysis. Interglob cells exhibit a preference for low-luminance or high-luminance stimuli over equiluminant stimuli. The histograms show AUCs for the ROC for each cell, for two luminance discrimination problems. An AUC <0.5 indicates a neuron preferred the first luminance in the pair; an AUC >0.5 indicates a preference for the second. Black bars indicate cells that significantly preferred one of the two luminance categories (*p* < 0.05, permutation test). The interglob population had significantly more cells whose AUCs were significantly different than chance than did the glob population, for both luminance discriminations (equiluminant/low-luminance: *p* = 0.00002, Fisher’s exact test, two-tailed; high-luminance/equiluminant: *p* = 2.6 × 10^−13^). ***B***, Luminance-invariant color tuning assessment in globs (left) and interglobs (right). For each cell, the peak angle within the low-luminance set is plotted against the peak angle within the equiluminant set (first and third columns), and the peak angle within the equiluminant set is plotted against the peak angle within the high-luminance set (second and fourth columns). The marker color indicates the hue to which the cell maximally responded for the *x*-axis stimulus set. The marker size increases with the cell's tuning narrowness. Because color space is circular, stimuli are rotated to appear at the *y*-axis point as close to the *x* = *y* line as possible. For example, if a cell had its peak at 10 CIELUV degrees for the equiluminant set and 355° for the low-luminance set, it would be plotted at (10, −5) rather than (10, 355). ***C***, Peak shifting histograms. Each cell was placed into one of eight bins based on the angle to which it maximally responded within a luminance level. Lighter bars show the peak tuning of the cells in each bin at the lighter of the two luminance values shown (equiluminant for the first and third columns, high-luminance for the second and fourth columns). Darker bars show the peak tuning of the same cells, at the darker of the two luminance values shown (low-luminance for the first and third columns, equiluminant for the second and fourth columns). The solid line shows the mean tuning angle for the higher of the two luminance values shown, and the dashed line shows the mean tuning angle of the cells at the darker of the two luminance values shown. Arrowhead designates the peak rod response hue angle.

The tuning curves of example cells (Fig. 2) suggest that the neurons in both the glob and interglob populations carry luminance information: in both sets of cells, the peak response amplitude and hue tuning of the neuron varied somewhat with changes in luminance level. We performed an ideal observer ROC analysis to quantify the luminance sensitivity of glob and interglob cells. Although implementations can differ (see Materials and Methods), the ROC analysis can be described as follows. For each neuron, we computed histograms showing the number of stimuli that elicited a given firing rate: one histogram for responses to the stimuli of the high-luminance set, one for responses to the equiluminant set, and one for responses to the low-luminance set. The ROC analysis compares the extent to which the histograms overlap: the less the overlap, the more likely the cell could distinguish the luminance difference between the stimulus sets. We performed two comparisons: equiluminant versus low-luminance sets; and high-luminance versus equiluminant sets (Fig. 9*A*). Given a criterion firing rate (selected from the range of firing rates produced by the neuron), we calculated the proportion of stimuli to which the first histogram exceeded the criterion, and the proportion of stimuli to which the second histogram exceeded the criterion. The calculation was performed for criterion values spanning the response range of the neuron; and we plotted the proportion of stimuli on which the second histogram exceeded the criterion as a function of the proportion of stimuli on which the first histogram exceeded the criterion. From these plots, we computed an AUC. Data points for histograms that perfectly overlap would fall along the diagonal (AUC = 0.5) and indicate that the neuron could not distinguish the luminance of the two sets of stimuli. AUC values <0.5 would indicate that the neuron could distinguish the luminance, and, moreover, that the neuron preferred the luminance associated with the first stimulus set. AUC values >0.5 would indicate that the neuron preferred the luminance associated with the second stimulus set.

Figure 9*A* shows AUC measurements for the population of neurons, for the three sets of comparisons. Black bars indicate neurons whose AUCs were significantly different from chance on permutation test^a^. On average, compared with the interglob population, the glob population contains fewer neurons that were capable of discriminating luminance (there are fewer black bars in the two leftmost plots). The glob and interglob populations contained different proportions of cells that could distinguish luminance, for each discrimination problem (equiluminant vs low-luminance: *p* = 0.00002^b^; and high-luminance vs equiluminant: *p* = 2.6 × 10^−13^.^b^ The glob cells that could discriminate luminance were equally distributed into ones preferring high-luminance, equiluminant, or low-luminance. By contrast, the majority of interglob cells showed a preference for the high-luminance or the low-luminance stimuli, but rarely the equiluminant stimuli: there is a greater proportion of cells with AUCs >0.5 for the low-luminance versus equiluminant comparison (Fig. 9*A*, third column), and <0.5 for the equiluminant versus high-luminance comparison (Fig. 9*A*, rightmost column). These results support the idea that the luminance response among the interglob cells does not contribute to the neural representation of color, whereas the luminance response among the glob cells does contribute to the neural representation of color.

We also tested the tolerance to luminance modulation of hue tuning in the globs and interglobs. Figure 9*B*, leftmost column, shows the peak tuning angle (Eq. 1) of each glob cell for the equiluminant stimulus set plotted against the peak tuning angle of the same cell for the low-luminance stimulus set. Figure 9*B* shows this analysis for equiluminant versus low-luminance globs (leftmost column), high-luminance versus equiluminant globs (second column), equiluminant versus low-luminance interglobs (third column), and high-luminance versus equiluminant interglobs (rightmost column). The glob cells showed higher peak angle correlation across luminance levels than the interglobs, for both the equiluminant versus low-luminance comparison (glob, *r* = 0.90; interglob, *r* = 0.80 equiluminant vs low-luminance, *p* = 1.27 × 10_−155_)^c^ and high-luminance versus equiluminant comparison (glob, *r* = 0.91; interglob, *r* = 0.84; high-luminance vs equiluminant, *p* = 4.70 × 10^−130^)^c^.

To determine whether or not peak shifting varied as a function of hue preference, we divided the glob and interglob cells into categories based on peak tuning preference. We defined eight bins of equal angle sizes (bin edges 0:45:360) and sorted each cell into a category on the basis of its peak color preference using the equiluminant stimulus set (Fig. 9*C*, left) or the color preference obtained using the high-luminance stimulus set (Fig. 9*C*, right). We then compared the color preferences obtained using the low-luminance (Fig. 9*C*, left) and equiluminant (Fig. 9*C*, right) stimulus sets for each category. This analysis was repeated for both glob and interglob populations, and for the equiluminant versus low-luminance and high-luminance versus equiluminant comparisons. In the high-luminance versus equiluminant comparison, cells were sorted based on their high-luminance peak angle. For the globs, only the bin containing orange-yellow cells (45:90) showed a significant peak shift according to a Mann–Whitney–Wilcoxon *U* test. The shift for this bin was significant for both the equiluminant versus low-luminance comparison (*p* = 0.009168, see Table 1 for a complete list of *p* values for hue categories)^d^, and high-luminance versus equiluminant comparison (*p* = 0.02754)^d^. For the interglobs, both the orange-yellow and yellow-green bins showed significant peak shifting, only for the high-luminance versus equiluminant conditions (orange-yellow, *p* = 0.009795; yellow-green, *p* = 0.02961)^d^. None of the eight hue bins showed significant hue shifting for the interglobs between equiluminant and low-luminance stimuli, despite the large differences in equiluminant and low-luminance mean peak hue angles (Fig. 9*C*, solid and dashed lines). This effect is likely due to the lower correlation in peak angle across luminance levels (Fig. 9*B*). Interglobs show large differences in peak hue tuning between luminance levels, but the shifts are inconsistent across cells: while most glob cells tuned to equiluminant yellow shift tuning such that they are tuned to low-luminance orange (Fig. 9*C*, leftmost column), some interglob cells tuned to equiluminant purple are tuned to low-luminance red, while others prefer low-luminance green and orange (Fig. 9*C*, third column). We did not find evidence of rod intrusion in this analysis: there was no systematic shift in peak tuning toward the peak rod sensitivity (Fig. 9*C*, black arrowhead) at lower luminance levels.

**Table 1:** Statistics

	Data structure	Type of test	Power [CIs]
aFigure 9A	N/APermutation test does not make assumptions about distribution of the data	Type of test: permutation test	*p* = 0.030 [0.0269–0.0334]
b Figure 9A		Description: test of whether there is a significant difference in the proportion of significant AUC cells in the glob vs interglob population (where significant AUC cells are those cells with AUCs significantly different than 0.5, or chance) for a given discrimination problem (e.g., low luminance/equiluminant)Type of test: Fisher’s exact test of proportions, two-tailedMethods used to compute 95% CIs: For each of 200 bootstrap samples of the cells (*N* = 300 for the globs, *N* = 181 for the interglobs), we determined the proportions of cells with significant AUCs and computed the test statistic described above. From the distribution of *p* values, we computed 95% CIs by the percentile method	Equiluminant/Low-luminance: *p* = 0.00002 [95% CIs: 3.95 × 10^−10^ to 0.006]High-luminance/Equiluminant: *p* = 2.6 × 10^−13^ [1.03 × 10^−21^ to 1.75 × 10^−8^]
c Figure 9B	Normally distributed after Fisher’s *z* transform	Description: Test of whether the Pearson’s *r* values quantifying the correlation between peak tuning angles at two luminance levels is significantly different for the glob and interglob populations. We performed 200 bootstraps to define a distribution of *r* values for each luminance population combinationType of test: Student’s *t* test (two-tailed) applied to the *z* transform of a bootstrapped distribution of *r* values.Methods used to compute 95% CIs: We performed 200 bootstraps 1000 times in order to get 1000 *p* values for each comparison. From the distribution of *p* values, we computed 95% CIs by the percentile method.	Significance of glob cell vs interglob cell luminance peak correlationEquilum vs Low Lum: *p* = 1.27 × 10^−155^ [6.32 × 10^−168^ to 1.50 × 10^−144^]High Lum vs Equilum: *p* = 4.70 × 10^−130^ [3.67 × 10^−141^ to 2.92 × 10^−118^]
d Figure 9C	N/AMann–Whitney *U* rank-sum test does not assume a normal distribution	Description: test for peak shifting between luminance levels for eight evenly sized color categoriesType of test: Mann–Whitney *U* rank-sum test (MATLAB ranksum)*Methods used to compute 95% CIs*: We performed the rank-sum test on 2000 bootstraps containing 90% of the cells in each population in order to get a distribution of 1000 *p* values for each comparison. From the distribution of p values, we computed 95% CIs by the percentile method	GlobEquilum vs Low Lum (in same order as in panel *C*, top to bottom): *p* = 0.1267 [0.1193–0.1342];0.4726 [0.4596–0.4857];0.3052 [0.2923–0.3182];0.2256 [0.2143–0.2369];0.2731 [0.261–0.2853];0.3197 [0.307–0.3324];0.009168 [0.007482–0.01085];0.3883 [0.3748–0.4018];High Lum vs Equilum: *p* =0.592 [0.5808–0.6031];0.4484 [0.4354–0.4614];0.1211 [0.1125–0.1296];0.4631 [0.4495–0.4766];0.4176 [0.4041–0.4311];0.5271 [0.5149–0.5394];0.02754 [0.02441–0.03067];0.4146 [0.4013–0.428]InterglobsEquilum vs Low Lum (in same order as panel *C*, bottom to top): *p* = 0.3541 [0.3409–0.3673];0.3396 [0.3259–0.3533];0.07011 [0.06317–0.07704];0.1924 [0.1805–0.2044];0.4253 [0.4117–0.439];0.312 [0.2988–0.3252];0.4446 [0.4312–0.458];0.152 [0.1417–0.1623];High Lum vs Equilum: *p* =0.4467 [0.4335–0.4599];0.1771, [0.166–0.1882];0.07582, [0.069–0.08263];0.4654, [0.4517–0.4791];0.1828 [0.172–0.1936];0.009795 [0.007742–0.01185];0.02961, [0.02585–0.03336];0.457 [0.4438–0.4702]

e Figure 11A	Normally distributed after Fisher’s *z* transform	Description: Test of whether a Pearson’s *r* quantifying the correlation between a CIELUV RDM and a neural RDM is significantly different from zeroType of test: Student’s *t* test (two-tailed) applied to the *z* transform of *r* Methods used to compute 95% CIs: For each of 200 bootstrap samples of the cells, we created an RDM, computed the correlation between this bootstrap RDM and the CIELUV RDM, and computed the test statistic described above. From the distribution of *p* values, we computed 95% CIs	*Significance of Glob and CIELUV RDM Correlation* High Lum set: *p* = 4.31 × 10^−296^ [95% CIs: 2.11 × 10^−316^, 6.63 × 10^−259^]Equilum set: *p* = 1.62 × 10^−271^ [2.82 × 10^−293^ to 3.76 × 10^−236^]Low Lum set: *p* = 3.71 × 10^−321^ [0 to 2.44 × 10^−276^]Significance of Interglob and CIELUV RDM CorrelationHigh Lum set: *p* = 3.33 × 10^−157^ [1.08 × 10^−171^ to 1.40 × 10^−114^]Equilum set: *p* = 5.16 × 10^−166^ [1.70 × 10^−174^ to 6.68 × 10^−115^]Low Lum set: *p* = 4.80 × 10^−93^ [2.03 × 10^−100^ to 3.31 × 10^−65^]Significance of Model LGN and CIELUV RDM CorrelationAll values are highly significant, with *p* < 2.23 × 10^−308^ (upper bound on 95% CI 0, equivalent to 2.23 × 10^−308^ in MATLAB 2015b)
f Figure 11A	Normally distributed after Fisher’s *z*-transform	Description: Test of significance of difference between glob and interglob Pearson’s *r* correlation coefficientsType of test: Paired *t* test (two-tailed) applied to *z*-transforms of *r’*sMethods used to compute 95% CIs: As described in d above, we computed correlations between bootstrap RDMs and the CIELUV RDM, but here, subsequently performed the test statistic described above.	High Lum set: *p* = 2.05 × 10^−8^ [95% CIs, 6.15 × 10^−5^ to 4.00 × 10^−15^]Equilum set: *p* = 1.70 × 10^−5^ [2.20 × 10^−3^ to 6.17 × 10^−11^]Low Lum set: *p* < 2.0 × 10^−16^ [2.22 × 10^−16^, < 2.0 × 10^−16]^
g Figure 11B	Classification accuracies; permutation test does not make assumptions about distribution of the data	Description: Test of whether classification accuracy is significantly above chance (50%)Type of test: Permutation testMethods used to compute 95% CIs: For each of 200 bootstrap samples, we determined a classification accuracy given the bootstrap sample, and performed a permutation test to obtain a *p* value. Since our null distribution contained 200 points, *p* was bound at 0.005, permitting calculation of only an upper 95% confidence bound.Because the glob and interglob populations were of different sizes, we subsampled the glob population to *N* = 181 for each of 200 subsample runs, and consider the ps for all subsamples	Hue decodingGlobsGeneralizing to High Lum: *p* < 0.005. [No null points lay above the observed decoding accuracy for any subsample of any bootstrap sample.]Generalizing to Equilum: *p* < 0.005. [No null points lay above the observed decoding accuracy for any subsample of any bootstrap sample.]Generalizing to Low Lum: *p* < 0.005. [No null points lay above the observed decoding accuracy for any subsample of any bootstrap sample.]InterglobsGeneralizing to High Lum: *p* < 0.005. [No null points lay above the observed decoding accuracy for any bootstrap sample.]Generalizing to equiluminant: *p* < 0.005 [upper 95% confidence bound = 0.08]Generalizing to Low Lum: *p* < 0.005 [upper 95% confidence bound = 0.315]Luminance decodingGlobsLow Lum/High Lum: *p* < 0.005. [No null points lay above the observed decoding accuracy for any bootstrap sample.]Low Lum/Equilum: *p* < 0.005 [No null points lay above the observed decoding accuracy for any bootstrap sample.]Equilum/High Lum: *p* < 0.005. [No null points lay above the observed decoding accuracy for any bootstrap sample.]InterglobsLow Lum/High Lum: *p* < 0.005. [No null points lay above the observed decoding accuracy for any bootstrap sample.]Low Lum/Equilum: *p* < 0.005. [No null points lay above the observed decoding accuracy for any bootstrap sample.]Equilum/High Lum: *p* < 0.005. [No null points lay above the observed decoding accuracy for any bootstrap sample.]

h Figure 11B	Binomial distribution	Description: Significance of difference between classification accuracies for the glob versus interglob populationsType of test: McNemar’s exact test, two-tailed on paired binomial data, with α = 0.05Methods used to compute 95% CIs: For each of 200 bootstrap samples, we obtained a p value by taking the average of 200 *p* values derived by comparing the results for the interglob population and the results for one subsampling run of the glob population using McNemar’s extact test, two-tailed (for paired binomial data).	Hue decodingGeneralizing to High Lum: *p* = 5.29 × 10^−7^ [95% CIs: 1.05 × 10^−10^ to 0.003]Generalizing to Equilum: *p* = 6.27 × 10^−12^ [1.88 × 10^−15^ to 1.12 × 10^−6^]Generalizing to Low Lum: *p* = 1.69 × 10^−5^ [1.88 × 10^−15^ to 0.006]Luminance decodingLow Lum/High Lum: *p* = 0.213 [95% CIs: 0.005–0.939]Low Lum/Equilum: *p* = 0.463 [0.003–0.570]Equiluminant/High-luminance: *p* = 0.074 [2.6 × 10^−6^, 0.375]
iDiscussion	N/A, permutation test does not assume a normal distribution	Description: significance of difference between proportion of warm tuned and cool tuned cellsType of test: permutation test. For 2000 permutations, each cell was randomly assigned one of the 45 stimulus angles to be tuned to. A null distribution of warm-tuned-to-cool-tuned cell ratios was calculated from this permutation.Methods used to compute 95% CIs: We ran 2000 bootstraps using 90% of each population. For each bootstrap, we calculated a *p* value as the proportion of permuted populations with a higher warm-tuned-to-cool-tuned cell ratio than the warm-tuned-to-cool-tuned cell ratio of the bootstrap population. The 95% CIs are calculated from the bootstrap populations. Because we had 2000 bootstrap permutations, the lowest bound of the *p* value possible is *p* < 5 × 10^−4^	GlobsHigh Lum: *p* = 0.001 [5 × 10^−4^ to 0.14]Equi Lum: 5 × 10^−4^ [5 × 10^−4^ to 0.001]Low Lum: *p* < 5 × 10^−4^ 5 × 10^−4^ to 5 × 10^−4^] (globs had higher warm-tuned-to-cool-tuned cell ratio than all permutations on all bootstraps)InterglobsHigh Lum: *p* = 0.0035 [5 × 10^−4^ to 0.15]Equi Lum: *p* = .51 [0.06–0.95]Low Lum: *p* = *p* < 5 × 10^−4^ 5 × 10^−4^ to 5 × 10^−4^]

Equilum, Equiluminant; High Lum, high-luminance; Low Lum, low-luminance; N/A, not applicable.

These results are consistent with the idea that the interglob population is sensitive to luminance contrast independent of the hue of the stimulus, whereas the glob population is sensitive to a combination of luminance contrast and hue. Such a combined sensitivity is predicted for neurons that represent a psychophysical color space in which the same hue at different luminance levels can be distinguished as having a different color. For example, orange and brown have the same hue but differ in luminance contrast. Certain glob cells would be capable of signaling both the hue and the luminance that distinguish orange from all other hues, and orange from brown.

### Quantitative comparison of population coding to color space

The tuning curves of example cells (Fig. 2) also suggest that both glob and interglob cells carry information about hue. In our next set of analyses, we considered how the globs and interglobs represent color information at the population level. We used MDS to first obtain a picture of neural color space, allowing our results to be compared with those of Namima et al. (2014). We then deployed RSA to quantitatively compare the neural representations of colors to the spatial relationships between those colors in CIELUV color space. To our knowledge, our analysis is the first in the single-unit literature that quantitatively tests the fit between neural representations and color space.

Each stimulus has a high-dimensional neural representation in which each dimension corresponds to the mean firing of one cell in the population of recorded neurons. For the glob population (*N* = 300), the representation of a given stimulus is 300-dimensional; for the interglob population (*N* = 181), it is 181-dimensional. From the high-dimensional neural representation, we can read out information about how similar the two stimuli are: we deem stimuli to be similar to the extent that their neural response vectors are correlated. MDS allows us to reduce (or “embed”) the high-dimensional neural representation to a lower-dimensional representation that we can plot, and that still captures (most of) the similarity structure of the original representation.

**Figure 10.**
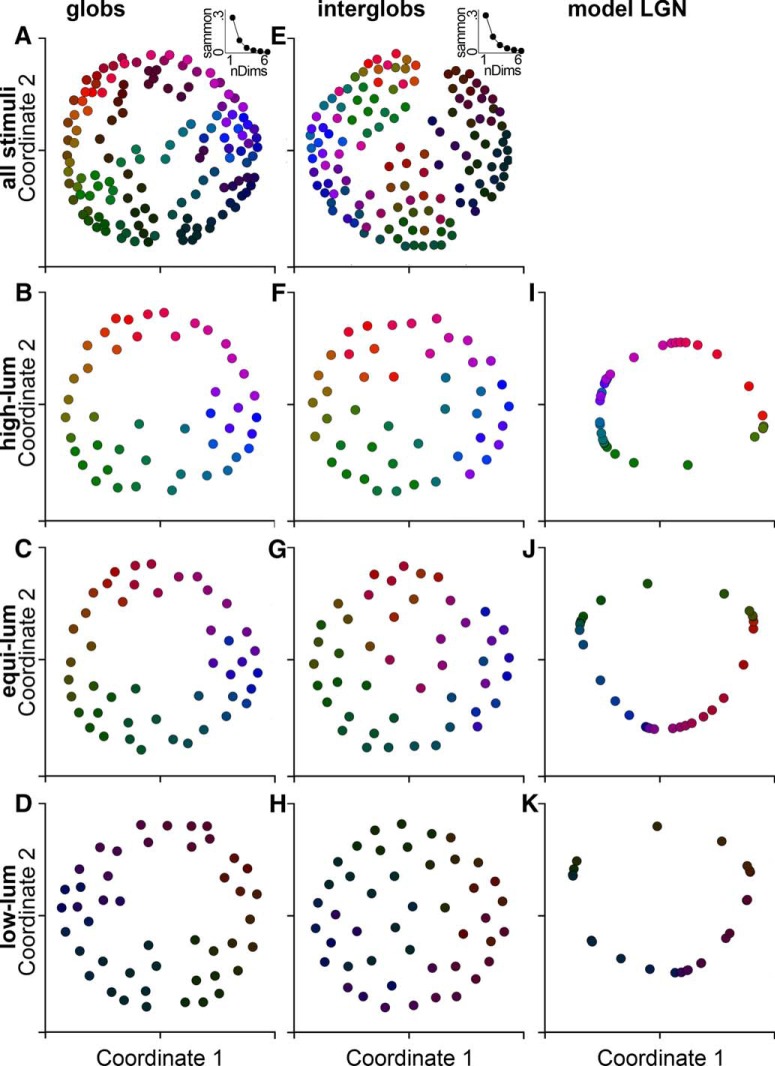
Glob, interglob, and LGN neural color spaces as calculated by MDS. Both the glob and interglob populations represent hue information, and these representations resemble the structure of perceptual CIELUV color space. ***A–K***, MDS applied to the glob (***A***, ***B***, ***C***, ***D***), interglob (***E***, ***F***, ***G***, ***H***), and model LGN (***I***, ***J***, ***K***) responses to stimuli yields a picture of neural color space. Each stimulus has a high-dimensional neural representation, where each dimension corresponds to the mean firing rate of a single cell. MDS produces a low-dimensional embedding that seeks to preserve the distances between stimuli in the original, high-dimensional space. We use 1 − ρ (Pearson’s correlation coefficient) as our distance metric: stimuli are distant to the extent that the patterns of activity they evoke are uncorrelated. Stimuli are plotted by their color in coordinates determined by the new, two-dimensional embedding. ***A***, ***B***, Glob and interglob MDS embeddings of the full stimulus set (45 hues at three luminance each). Insets show Sammon’s stress as a function of embedding dimensions used. Glob (***B–D***), interglob (***F–H***), and model LGN (***I–K***) MDS embeddings of stimulus hues for each luminance level (Sammon’s stress for globs: low-luminance = 0.05; equiluminant = 0.06; high-luminance = 0.05; for interglobs: low-luminance = 0.12; equiluminant = 0.10; high-luminance = 0.10; for model LGN cells: low-luminance = 0.10; equiluminant = 0.10; high-luminance = 0.10).

We can compute an error measure (we used Sammon’s stress) to determine how much the similarity relationships between pairs of points in the high-dimensional representation are uncaptured by a best-fitting lower-dimensional representation (Fig. 10, insets, top panels). While higher numbers of dimensions always produce better dissimilarity approximations, this comes at the cost of increased model complexity; for our embeddings, stress drops for two dimensions, and then decreases gradually thereafter. Figure 10 shows plots of the two-dimensional MDS embeddings for the two populations of neurons in V4/PIT, as well as a population of model LGN cells. The color of each data point corresponds to the color of the stimulus, and the spatial relationship of the points reflects the extent to which the neural responses to the stimuli were related: data points are plotted closest together when the neural responses to the two stimuli were most similar (see Fig. 12 for the three-dimensional MDS embeddings).

For the glob population, the arrangement of the stimuli clearly reflects CIELUV color space: points of the same hue irrespective of luminance level are plotted next to each other, and the progression of the points forms a circle that proceeds according the color wheel: following clockwise, blue is next to cyan, which is next to green, followed by yellow (brown), orange, red, and, closing the circle, purple. The pattern obtained for the interglob cells also bears some similarity to perceptual color space, but is not as clearly organized (e.g., cyan dots are intermingled with blue and purple dots).

The MDS analyses in the top panels of Figure 10 were performed using the neural responses to all the stimuli at all luminance levels. How do the populations represent hue information when luminance is removed as a variable? In other words, to what extent is the neural representation of color preserved across luminance levels? To answer this question, we performed MDS separately on responses to stimuli in each luminance set. For the globs, the representation of the stimuli largely reflects perceptual color space for all luminance levels tested (Fig. 10, *B-D*). For the interglobs, the representation of the stimuli corresponds less clearly to perceptual color space, especially when assessed using the equiluminant and low-luminance sets (Fig. 10, *F-H*). The MDS analysis suggests that behavioral judgments of the similarity between colors closely match the similarities between the neural responses to these colors by the glob population, and, to a lesser extent, by the interglob population. For comparison, we performed the same MDS analysis on a simulated population of parvocellular LGN neurons (see Materials and Methods). The MDS representation recovers the sequence of colors found in perceptual color space (Fig. 10, right column) but is distorted toward the stimuli that had the highest saturation, as expected from a population of neurons with linear tuning (Fig. 4).

If the sensitivity to color is reflective of a causal role in color perception, we hypothesized that the relative response of a population to pairs of stimuli should reflect the similarity of the colors of the stimuli defined by a uniform perceptual color space. To test this idea quantitatively, we performed RSA, in which we looked at the extent to which the representational dissimilarities between stimuli according to neural response are correlated with the dissimilarities according to perceptual CIELUV color space hue angle. Given any two stimuli, we can measure their dissimilarity both by the angular distance between their hues in CIELUV space, and by the correlation distance between their neural response vectors. Performing these two computations for each pair of stimuli, we obtain two 45 × 45 dissimilarity matrices, where 45 is the number of hues. To assess whether the two measures induce similar representations, we calculate the Pearson’s correlation coefficient between the two dissimilarity matrices. A high correlation indicates that the neural responses agree well with the color space.

To focus on hue, we split the stimuli into the three luminance sets (low-luminance, equiluminant, and high-luminance), and for each luminance set, we performed RSA on each of the glob and interglob population responses. Given our MDS results (Fig. 10), we predicted that the glob representations of hue at each luminance would strongly correlate with CIELUV hue space for all luminance sets, but that for the interglobs this correlation would be high only for the high-luminance set. Further, we expected there to be a significant difference in the correlation values for the two populations.

**Figure 11.**
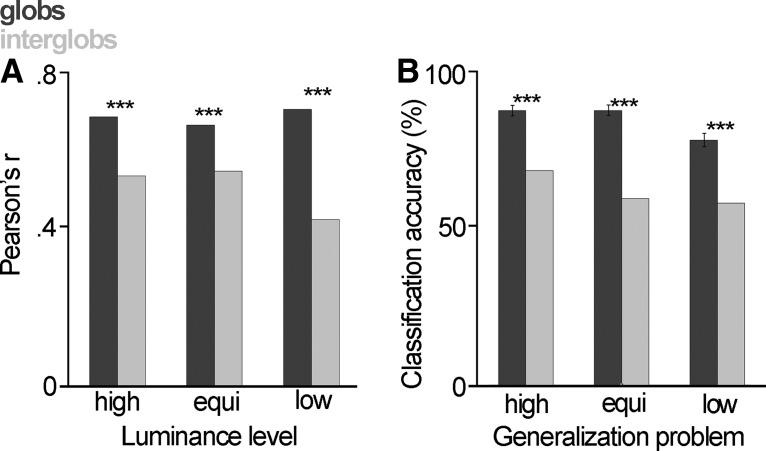
Neural color space and CIELUV hue RSA (***A***) and hue decoding (***B***). ***A***, Neural distances between stimuli are significantly correlated with the hue angle distances between these stimuli in perceptual CIELUV color space. Both glob and interglob population representations are significantly correlated with CIELUV for the high-luminance, equiluminant, and low-luminance stimulus sets (*p* < 0.0001, *t* test, two-tailed). For all sets, the glob representations are significantly more correlated with CIELUV than are the interglob representations (high-luminance set: *p* = 2.05 × 10^−8^, *t* test two-tailed, Fisher r-to-z; equiluminant set: *p* = 1.70 × 10^−5^; low-luminance set: *p* < 2.0 × 10^−16^). ***B***, Representations of the same hue at different luminance levels are sufficiently similar that hue information can be read out by a linear SVM invariant to changes in luminance. Mean pairwise hue classification accuracies for three generalization problems. A classifier was trained to distinguish between hues given two stimulus sets (e.g., low-luminance and equiluminant), and tested on the held-out stimulus set (e.g., high-luminance). The test luminance appears below each set of bars. Classification accuracy was significantly above chance for all generalization problems in both populations (*p* < 0.005, permutation test), though significantly higher for the glob than interglob population (all generalization problems: *p* < 0.0001, McNemar’s exact test, two-tailed). Error bars indicate the SD of the mean across 200 subsampling runs.

As shown in Figure 11*A*, we found that the representations by the glob population of the low-luminance, equiluminant, and high-luminance sets were indeed significantly correlated with the angular distances between stimuli (high-luminance set: Pearson’s *r* = 0.70, *p* = 4.31 × 10^−296^, two-tailed *t* test^e^; equiluminant set: *r* = 0.68, *p* = 1.62 × 10^−271^; low-luminance set: *r* = 0.72, *p* = 3.71 × 10^−321^). The representations of the interglob population were also significantly correlated for each set (high-luminance set: *r* = 0.55, *p* = 3.33 × 10^−157^; equiluminant set: *r* = 0.56, *p* = 5.16 × 10^−166^; low-luminance set: *r* = 0.43, *p* = 4.80 × 10^−93^). But the glob representations were more similar to CIELUV than were the interglob representations (high-luminance set: *p* = 2.05 × 10^−8^, paired two-tailed *t* test^f^; equiluminant set: *p* = 1.70 × 10^−5^; low-luminance set: *p* < 2.0 × 10^−16^), showing that hue information in the glob population more closely resembles perceptual color space. We performed the same analysis on a population of model LGN cells and, similarly, found high correlations between their representation of the stimulus sets and the angular distances between stimuli: (high-luminance set: *r* = 0.83; equiluminant set: *r* = 0.96; low-luminance set: *r* = 0.89; all correlations were significant, *p* < 2.23 × 10^−308e^, two-tailed *t* test).

### Decoding of hue information across changes in luminance

Contemporary color-ordering systems treat hue and luminance as separable parameters. To examine whether the extraction of hue and luminance from colored stimuli could be supported by the neural data, we used invariant decoding: we used a pattern classifier to predict the hue of a stimulus from its neural representation. During training, we presented the classifier with labeled examples of the neural response vectors for two hues at two luminances each (e.g., low-luminance green, equiluminant green, low-luminance blue, equiluminant blue). We then tested the classifier by asking it to predict the hues of two new neural response vectors, where our test cases were the same two hues seen during training, but at a new luminance (e.g., high-luminance green and high-luminance blue). To do well on this task, then, our classifier must generalize the hue information it learned during training to correctly classify these hues at a luminance it has never seen before. We obtain a comprehensive measure of classification accuracy by averaging the results for each pair of hues. High classification accuracy indicates that the population represents hue in such a way that it is sufficiently similar as to be recognizable across changes in luminance.

When a classifier successfully “reads out” an experimental variable given a pattern of activity, this suggests that the information may be accessible to upstream neurons. Similar analyses have been used to look at how neural populations represent object identity invariant to changes in position and scale (Hung et al., 2005; Rust and diCarlo, 2010) and to recover color information from fMRI data (Brouwer and Heeger, 2009). Given that our RSA and MDS results showed that the glob population captures hue information to a greater degree than does the interglob population, we predicted that classification accuracy would be higher for the glob cells.

Figure 11*B* shows results for both populations for three classification problems, namely, problems in which we (1) train on low-luminance and equiluminant sets, and test on high-luminance; (2) train on low-luminance and high-luminance, and test on equiluminant; and (3) train on equiluminant and high-luminance, and test on low-luminance. Classification accuracy was significantly above chance for all three problems for both the glob and interglob populations (all *p* < 0.005^g^, permutation test). But classification accuracies were significantly higher for the glob than the interglob population (generalizing to high-luminance: *p* = 5.29 × 10^−7^, two-tailed McNemar’s exact test^h^; generalizing to equiluminant: *p* = 6.27 × 10^−12^; generalizing to low-luminance: *p* = 1.69 × 10^−5^).

### Analyses of luminance information present in the population responses

**Figure 12.**
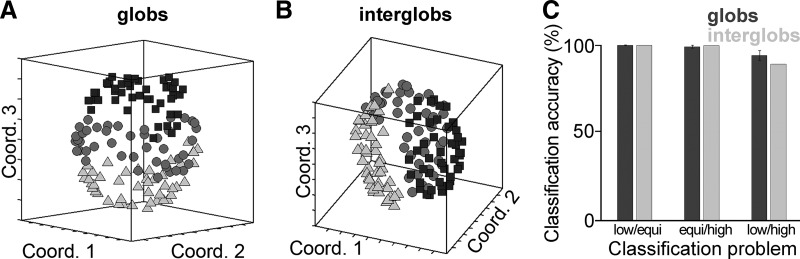
Luminance clustering within neural color space (***A***, ***B***) and luminance decoding (***C***). Both the glob and interglob populations segregate stimuli by luminance. ***A***, ***B***, Three-dimensional MDS embedding for the glob (***A***) and interglob (***B***) populations. Squares indicate low-luminance stimuli, circles indicate equiluminant stimuli, and triangles indicate high-luminance stimuli. ***C***, Luminance information is accessible to readout by a linear classifier invariant to changes in hue for both populations, showing that these populations carry information about stimulus luminance as well as hue. Mean classification accuracies across decoding runs were high (>89%) for distinguishing between all luminance class pairs, for both populations (all *p* < 0.005, permutation test). Error bars indicate the SD of the mean across 200 subsampling runs.

In Figure 9, we showed that many of our individual cells can discriminate stimulus luminance. How does this ability manifest at the population level? As we did with hue, we can use MDS embeddings to visualize stimuli by luminance category. Figure 12 shows the three-dimensional MDS embeddings for the glob (Fig. 12*A*; Sammon’s stress = 0.03) and interglob populations (Fig. 12*B*; stress = 0.06). In both populations, the stimuli are well segregated by luminance. Striking is the fact that, in the globs, each luminance set forms a discrete ring, and each of these rings contains a full hue map, similar to three-dimensional renderings of CIELUV color space. Movies 1 and 2 show the rotations of the three-dimensional embedding for the glob population colored by stimulus color (1) and luminance category (2). Movies 3 and 4 show the rotations of the interglob embedding.

Movie 1. Three-dimensional MDS stimulus embedding for the glob population, colored by stimulus color. Rotations reveal hue-organized rings. 10.1523/ENEURO.0039-16.2016.video.1

Movie 2. Three-dimensional MDS stimulus embedding for the glob population, colored by luminance category. Squares indicate low-luminance stimuli, circles indicate equiluminant stimuli, and triangles indicate high-luminance stimuli. Rotations reveal three luminance rings. 10.1523/ENEURO.0039-16.2016.video.2

Movie 3. Three-dimensional MDS stimulus embedding for the interglob population, colored by stimulus color. 10.1523/ENEURO.0039-16.2016.video.3

Movie 4. Three-dimensional MDS stimulus embedding for the interglob population, colored by luminance category. Squares indicate low-luminance stimuli, circles indicate equiluminant stimuli, and triangles indicate high-luminance stimuli. Rotations reveal that the population segregates stimuli by luminance. 10.1523/ENEURO.0039-16.2016.video.4

Given the luminance category segregation apparent in the MDS plots, we expected that hue-invariant luminance decoding should be possible for both populations. Proceeding analogously to our hue-decoding analysis, we trained a classifier to distinguish between each pair of stimuli of constant hue and different luminance. For each pair, we trained the classifier by showing it luminance-labeled examples of 15 of 17 hues. Next, we tested its ability to predict the luminance of the two hues that had been left out. We found that the classifier was able to read out luminance information with high accuracy invariant to changes in hue for both neural populations (>89%, *p* < 0.005, permutation test^g^; Fig. 12*C*). Classification accuracies were not significantly different between the glob and interglob populations.

## Discussion

The organization of colors can safely count as one of the longest-lived problems in science (Kuehni and Schwartz, 2008). Newton was perhaps the first to systematically arrange colors in a circle, making meaningful use of geometry; his insight suggested that color organization was determined not solely by physical parameters but also by the way signals are processed in the brain. Contemporary color-ordering schemes adopt three dimensions: hue (e.g., “red,” “purple,” “green”), saturation (“red” vs “pink”), and brightness (or value). Unknown are the neural rules that determine the geometric relationships within and between these dimensions. One tradition proposes that color is organized around six unique hues (Hering, 1905; Hurvich, 1981), which were initially thought to reflect color tuning in the LGN (De Valois et al, 1966). The psychological importance of these colors is not unquestioned (Saunders and van Brakel, 1997; Witzel et al., 2015; Wool et al., 2015). And careful analysis shows that LGN color tuning does not underlie the unique hues (Webster et al., 2000); the neural basis for the unique hues remains unknown. Where Newton’s color-ordering system was launched by physics, and Hering’s color-opponent scheme began with psychology, our approach starts with the structural organization of perception and the representation of color in the brain. Our goal is to determine what brain areas (and cells) represent color, and to interrogate the neural responses to reverse engineer the rules that govern color space geometry. We have not yet reached this goal, but here we present evidence showing that a population of neurons (glob cells) in PIT/V4 not only contains a representation of color space that bears remarkable similarity to uniform perceptual color space, but also possesses nonlinear (narrow) color tuning, two features that suggest an important role in color perception.

Color-tuned neurons have been found earlier than V4/PIT in the visual-processing hierarchy. V1 has received considerable attention (Gegenfurtner and Kiper, 2003; Solomon and Lennie, 2007; Conway, 2009; Conway et al., 2010; Shapley and Hawken, 2011), although linear systems analysis has not uncovered a meaningful correspondence between V1 color-tuning properties and color space (Lennie et al., 1990). An analysis of just the population of cone-opponent V1 cells—cells most likely involved in color processing—uncovered an over-representation of the colors associated with daylight (Conway, 2001; Lafer-Sousa et al., 2012), possibly providing a Bayesian prior used to resolve stimulus ambiguity (Lafer-Sousa et al., 2015). Other work, applying a nonlinear analysis, suggests that V1 cells inherit the chromatic-tuning biases of the LGN (Horwitz and Hass, 2012).Together, this research suggests that V1 constitutes an intermediate step in the computation of color. Consistent with this hypothesis, imaging in humans shows that the color space derived from the covariation across V1 voxels in response to different colors does not correspond to perceptual color space; instead, higher-order areas (hV4, V-O1) possess a representation that more closely corresponds to perception (Brouwer and Heeger, 2009). Cells carrying color information are found in V2 (Burkhalter and Van Essen, 1986; Hubel and Livingstone, 1987; Moutoussis and Zeki, 2002); as a population, V2 cells show a bias for colors of daylight (Kiper et al., 1997), similar to V1. One study suggests that V2 contains maps that match color space (Xiao et al., 2003; Lim et al., 2009), but quantitative tests of this correspondence have not been performed. Neurons in V3 either appear indistinguishable in their color-tuning properties from V2 neurons (Gegenfurtner et al., 1997) or they carry considerably less color information (Baizer, 1982).

A substantial transformation of color signals takes place in or before area V4/PIT (Zeki, 1980; Desimone et al., 1985). The importance of V4 in color processing was initially challenged (Schein et al., 1982), but the controversy was resolved with experiments combining fMRI and fMRI-guided microelectrode recording, which showed that V4 contains color-biased subdomains, “globs,” that are separated by interglob regions showing lower color bias (Conway and Tsao, 2006; Conway et al., 2007). These findings have been confirmed using optical imaging (Tanigawa et al., 2010; Li et al, 2014). Prior work suggests that glob cells have narrow color tuning (Conway et al., 2007), but the narrowness has not been quantified until now. Here we show that glob cells are probably much narrower in their tuning than LGN cells (and most V1 cells). The stimuli used presently, which are typical of many neurophysiological studies of color, preclude a definitive conclusion because they confound saturation and hue. Nonetheless, the likely narrow tuning, coupled with the representation of color space encompassed by the population of glob cells, suggests a neural basis for higher-order psychophysical chromatic mechanisms (Webster and Mollon, 1991; Hansen and Gegenfurtner, 2007; Stoughton et al., 2012).

A preliminary analysis suggested that the glob cell population is biased toward the unique hues (Stoughton and Conway, 2008). The stimuli used to obtain these data were similar to stimuli used in other studies (Komatsu et al., 1992): they consist of the most vivid colors permitted by the monitor. These colors lie on a triangle in CIE space. Cone contrast varies among stimuli within this set, and is highest for the red and blue apices. It has been suggested that the color-tuning biases observed in the population of glob cells would be observed in the LGN, if LGN responses were assessed with the triangular stimulus set (Mollon, 2009). Alternatively the color-tuning distribution may reflect the responses of a population that has color-tuned neurons uniformly representing color space, in which each neuron is sensitive to saturation (Conway and Stoughton, 2009). We tested these alternative predictions by comparing model simulations to recorded data. Simulations that assume linearly tuned neurons did not match the glob cell data: the simulations only yielded peaks to colors located at the apices of the triangle, unlike the measured neural population that also had peaks at intermediate colors. These findings rule out the first possibility: glob cells do not appear to have color tuning as found in the LGN. Among nonlinear models, does the best-fitting model show a bias toward the cardinal directions? It seems not. The nonlinear models biased to the cardinal directions showed too few responses to green compared with the glob cell data. Instead, the optimal model was a uniform representation. Among glob cells, those tuned to purple showed the narrowest color tuning, a feature that may reflect the peculiar properties of S cone-opponent neurons in V1 (Cottaris and De Valois, 1998; Conway and Livingstone, 2006; Horwitz and Hass, 2012), LGN (Tailby et al., 2008b), and retina (Dacey and Lee, 1994). These cells probably account for the band corresponding to models with a tuning width of 96 (Fig. 6). The nonlinear models biased for the unique hues did not fare any better at each level of nonlinearity than the models of populations biased to the cardinal colors: the best model was still close to a uniform distribution. These results suggest that the variation in saturation among the stimuli is the cause of the bias toward red, green, and blue that was reported previously.

The best-matching models in all the comparisons have *R* ^2^ values hovering around 0.5, leaving plenty of variation unaccounted for. Some of this variability may be attributed to uneven sampling of neurons. It is also possible that the color space sampling, every ∼17**°**, was too sparse; we suspect this is not the case because we did not find systematic changes of narrowness as a function of color tuning when using stimuli that more finely sample color space. The stimuli were all relatively low luminance, raising the possibility of rod intrusion (Stiles and Burch, 1959). The analysis of color responses at different luminance levels does not indicate a bias predicted by the rod peak, although the impact of rod intrusion could be complex (Shapiro et al., 1996; Buck, 1997). The complications of interpreting the neurophysiological data described here underscore the critical importance of the careful choice of stimuli, and draw attention to the overlooked gaps in our understanding of what constitutes seemingly elementary properties of color (hue, saturation, and brightness) and their interaction. In particular, we note that there remains no consensus on the spatial organization of color space: CIELUV, the perceptual space used here, is a standard, but it has defects (Melgosa et al., 1994)

Prior work has shown that glob cells are spatially clustered by color preference (Conway and Tsao, 2009). Conway and Tsao (2009) present the first microelectrode evidence for chromotopic maps anywhere in the visual system, but they perform no quantitative tests of the correspondence between the neural representation of color and the organization of perceptual color space. The data presented here fill this gap, and strongly suggest that the population of glob cells contains narrowly tuned neurons representing most directions in color space, not just the cardinal directions favored by the LGN. Moreover, using representational similarity analysis, we found that the representation of color space encoded by the glob, as compared with the interglob, population, shows a better correspondence to CIELUV color space; the glob cell population also shows a bias toward warm colors (reds, yellows). RSA of a model LGN population also showed similarity to CIELUV color space, which demonstrates that a correspondence with color space can occur in the absence of single cells narrowly tuned to each color in space. The RSAs on the two cortical populations suggest that between glob and interglob populations, it is the glob cells that perform the readout of the color space representation found in the LGN. This conclusion is supported by the narrow tuning found among glob cells and the decoding algorithms showing that stimulus color can be predicted from the color tuning of the most active neurons in the glob cell population (Zaidi et al., 2014).

The responses to the luminance of the different sets of stimuli, by both glob and interglob populations, could be read out by a linear classifier. But a linear classifier decoded the hue invariant to luminance better for the glob than for the interglob population. Luminance classification accuracy (luminance invariant to hue) was high for both populations. One might have thought that neurons implicated in color perception (glob cells) would show color tuning that was entirely invariant to changes in luminance. But luminance is an important dimension of color, and can change a hue from one color into another: an increase in luminance contrast (brightness) converts brown into orange. The sensitivity to luminance contrast of the glob cells is consistent with prior observations (Conway et al., 2007; Namima et al., 2014), and may underlie the Bezold–Brucke hue shift (Stoughton and Conway, 2008). The interglob cells, meanwhile, typically did not retain their hue tuning across luminance levels. We quantified this result, and confirm that interglob cells tended to show clear preferences for stimuli that were either darker or brighter than the background, regardless of hue. Together, the results on the interglob cells suggest that this population is using chromatic information in the service of something besides color perception, such as the detection of color boundaries for object recognition. Such a computation would be useful in object segmentation and in defeating camouflage, where sensitivity, but not selectivity, for color is crucial; this property of interglob cells suggests that they are part of the network that includes the “complex equiluminance” cells found in V1 (Conway and Livingstone, 2006; Conway, 2014).

Imaging experiments show that considerable cortical territory within and anterior to V4 is implicated in color (Tootell et al., 2004; Conway et al., 2007; Harada et al., 2009; Lafer-Sousa and Conway, 2013); there is a high degree of homology in the functional organization of the ventral visual pathway in humans and IT cortex in monkeys (Wade et al., 2008; Lafer-Sousa et al., 2016). Microelectrode recording and anatomical track-tracing experiments in monkeys confirm that extrastriate regions identified as color biased using fMRI typically are enriched for color-tuned neurons (Conway et al., 2007) and probably connected to each other (Banno et al., 2011). Namima et al. (2014) offer the first systematic comparison of the narrowness of color tuning at multiple stages spanning the temporal lobe, from V4 to AIT. They found that neurons in AIT showed more nonlinear tuning than neurons in V4/PIT, although they did not target recordings to color-biased domains identified independently with imaging. We wondered whether the relatively narrower tuning of neurons in AIT might be evident in V4/PIT, if one restricted the analysis to just globs. If so, then the high nonlinearity in AIT might not be generated cumulatively through serial stages along IT, as suggested by Namima et al. (2014), but rather created early in processing and inherited by AIT. Consistent with our hypothesis, glob cells had considerably narrower color tuning than interglob cells. Indeed, the narrowness of the glob cells was higher than the estimates for neurons in AIT (93% sharply selective glob cells, compared with ∼75% in AIT, as estimated by Namima et al., 2014).

The results presented here suggest that the neural correlate of perceived color is computed in V4/PIT. What then is the functional role of the vast amount of color-biased cortical tissue within the rest of IT? At this point, we can only speculate. Chromatic information informs many behaviors ranging from attentional recruitment, to memory, to social cognition (Conway, 2016).These behaviors underscore an important advantage of color perception: the system is trainable and can forge associations not only between colors and objects, but also between colors and abstract concepts such as emotional states (red/anger) and words. IT is implicated in many aspects of high-level object vision, including recognition, categorization, memory, and attention. Koida and Komatsu (2007) have shown that the firing of color-tuned neurons within AIT is influenced by task demands, lending support to the idea that the job of more anterior regions of IT is not to compute color perception (a job taken care of by PIT), but rather to use this information to direct behavior. It seems likely, then, that the color-biased regions within AIT are involved in high-level behaviors that depend on or involve color.

Hering (1964) made a powerful case that opponency is the basis for color vision. He took this observation further to argue that color is constructed by three sets of exclusive color pairs: red/green; blue/yellow; and black/white. The results from the study by Hering (1964) have been interpreted as supporting the theory that basic color categories are universal and derive from the hardwiring of color tuning in the nervous system (Berlin and Kay, 1969; Lindsey and Brown, 2009). But the universalist theory has received some hard blows challenging the methodology (Saunders and van Brakel, 1997). Psychophysical work has also challenged the theory: the unique hues are not more salient than other colors, as they should be if they are privileged (Wool et al., 2015). Moreover, there is considerable variability among people (Webster et al., 2002), and across language groups (Lindsey et al., 2015), in the location and boundaries of the unique hues within color space; and the specific colors that are assigned special status vary across cultures (Davidoff et al., 1999; Roberson et al., 2000), which suggests that the unique hues are not as special as widely assumed. Neurophysiological work strongly suggests that the building blocks of color processing in the retina and LGN depend on cone opponency (Conway, 2009); but this opponency does not account for the unique hues or basic color categories (Webster et al., 2000). Is it possible that the importance of the unique hues is learned, reflecting some special behavioral importance, and as such depends on processing in higher-order areas, rather than being innate and accounted for by activity early in the visual-processing hierarchy? The ultimate goal of the visual system is to transform an inherently ambiguous retinal stimulus into an unequivocal signal that can guide action. The retinal stimulus for color is typically ambiguous, which seems to be at odds with our experience of color: most observers are under a powerful illusion that color (including the importance of the unique hues) is tied in some direct way to the physical world (Conway, 2009, 2016)—the apparent unequivocal nature of our experience of color is one of the triumphs of the brain. Each of us has great conviction about the accuracy and validity of our color experience, even when there is no clear consensus among people considering the same stimulus (Lafer-Sousa et al., 2015). Our convictions could corrupt experimental design: in retrospect, it seems likely that the methods of the important World Color Survey (http://www1.icsi.berkeley.edu/wcs/) begged the existence of basic color terms (Saunders and van Brakel, 1997). Rather than reflecting a fundamental input to the color vision machinery, the unique hues more likely represent the computational product of this machinery, reflecting the integration of task demands, behaviorally relevant statistics, and language, computations that we hypothesize are implemented in IT and the frontal cortex (Lafer-Sousa and Conway, 2013; Romero et al., 2014). These arguments reformulate the question regarding the unique hues: do they have some special behavioral relevance that underlies their privileged status?

Throughout this article, we have referred to “color-biased regions” rather than “color areas.” We draw this distinction because we are not yet in a position to conclude what computations these regions are performing. The term “color area” implies a specific and exclusive role in computing color. Given the extensive amount of cortical real estate in the temporal lobe that responds to chromatic stimulation, the wide range of operations that could benefit from sensitivity to color, and the likely sensitivity of color-biased regions to other stimulus attributes such as texture and material properties (Goda et al., 2014), it would seem premature (and wrong) to conclude that color-biased regions, including the globs of V4/PIT, are functionally discrete areas dedicated to processing only color. The analysis presented here provides a method for quantitatively testing the relationship between color behavior and neural activity that we hope to exploit in tracing the transformation of color signals through these regions to figure out what the regions are doing and how color tuning emerges. The close relationship between perceptual color space and the neural representation of color found among glob cells of PIT/V4 shows what information is available to subsequent stages in the putative visual-processing hierarchy, and will guide future work measuring neural activity with stimuli that densely sample uniform color space in an attempt to find a solution to the geometry of color space.

## Synthesis

The decision was a result of the Reviewing Editor Tim Bussey and the peer reviewers coming together and discussing their recommendations until a consensus was reached. A fact-based synthesis statement explaining their decision and outlining what is needed to prepare a revision is listed below. The following reviewer(s) agreed to reveal their identity: Qasim Zaidi

The reviewers and I felt the MS was very well written, with an impressive amount of analysis of colour responses of PIT cells, much of it systematically done. The paper carefully considers some important questions in color theory, and the results are likely to be of significant interest to the field.

As pointed out by one reviewer in particular, all of the analyses rely on the measurements being tuning curves, and it was felt that this needed more justification. As this reviewer put it: "The original measurements were made to 45 colors along the triangle formed by the three phosphors of a color monitor. The authors try to approximate tuning curves by sampling test colors to be roughly equally spaced in the CIELUV diagram. There is not sufficient justification of this assumption in the paper. As far as I know, even industry people only consider CIELUV to be a locally uniform space, and even that is not based on any data more recent than MacAdam's color matches. It would be useful to see references to empirical evidence that CIELUV is a uniform space for color differences as big as the ones used in this study. Even with this assumption, the sampling is 22.5 deg around the color circle. That would be fine for cosine-tuned cells, but is unlikely to provide good estimates for narrowly tuned cells."

At the same time this same reviewer felt that an argument could be made that the Conway data is the most complete existing characterization of the color responses of PIT cells, and without a huge amount of data collection, it is not possible to improve on CIELUV as a uniform space.

The reviewers ask you to consider a number of additional points in your revision:

1. "The colors of one set were higher luminance (7.8 cd/m2) than the background; those of another set were photometrically equiluminant with the background (3.05 cd/m2); and those of the third set were lower luminance than the background (0.62 cd/m2)." A number of standard sources do claim 3cd/m2 as the lower limit of the photopic regime, but it has been repeatedly shown that even at low photopic levels, for fields covering more than the central fovea, there is rod intrusion in color matching, e.g. the failure of scalar invariance in the Stiles 10 degree CMFs. Hence for each of these luminance levels, the authors need to show that rod intrusion does not play a part in distorting responses across the color space. A simple way to test this is to assume ceteris paribus that there should be more rod intrusion at the lower levels. That would predict a systematic shift in the tuning curves from high to low levels. The nature of the shift could be derived from published calculations based on CRT phosphor spectra (e.g. Shapiro AG, Pokorny J, Smith VC. Cone-rod receptor spaces with illustrations that use CRT phosphor and light-emitting-diode spectra. JOSA A. 1996 Dec 1;13(12):2319-28.). If that shift does not occur, then there is less reason to be worried about rod intrusion.

2. Please explain how: "Using stimuli at different luminance levels provides one way of controlling for luminance artifacts arising from, for example, chromatic aberration or variability in macular pigmentation."

3. "To do so, we subsampled the data, extracting responses to 16 hues (at each luminance level) that were relatively evenly spaced in perceptually uniform CIELUV (C.I.E., 2004) angle". What is the evidence that CIELUV is a uniform color space? My understanding is that it is just a linear correction to CIELAB, which was based on MacAdams color-matching errors, not thresholds, and certainly did not address uniformity of even moderate size color differences. I am raising this point because it is very important in evaluating the results of this paper, since the interpretation of the recordings as tuning curves depends critically on whether the colors are equally spaced on the important metric. Also were a total of 16 hues used for all the cells? Isn't this too sparse a set for narrowly tuned neurons, essentially 22.5 deg apart in angle.

4. Even if the hues were equally spaced, their saturations need to be equated.

"We defined saturation in DKL space". This would rely critically on the relative scaling of the axes. How was this done? Were any measurements made to show equal saturations for equal excursions from gray in different directions? Why use DKL for saturation and not CIELUV?

5. "The time window began with the visual latency, which was defined as >2.5 standard deviations above the background firing rate, and ended when the response rate fell below the background firing rate + 2.5 standard deviations." Are all responses increases in firing rate, and no responses decreases?

6. For fitting narrow tuning curves, there are much better alternatives than the DeValois equation, in which the exponent controls the slope near threshold as in this equation, but a divisor inside the sine function controls the width.

7. "We chose to curve fit responses to only the 16 evenly-spaced stimuli" Evenly spaced in what metric?

8. "To account for differences in saturation among the stimuli, the model cell's response to each stimulus was multiplied by that stimulus's saturation." To justify this adjustment, the authors would have to show that the contrast response curve is linear, and that is highly unlikely.

9. "To test whether the populations are best described by a distribution biased towards the cardinal directions, by the unique hues, or alternatively by a uniform distribution, we compared the neurophysiological results to model predictions." Doesn't this comparison require random sampling? How close to that assumption was the actual data collection?

10. "Figure 2 shows  tuning curves obtained for representative cells, in the globs (panel A) and interglobs (panel B).  Glob cells typically showed narrower color-tuning curves than interglob cells, and color  preferences that were retained across different luminance levels." This results is both interesting and incomplete. Does it mean that both glob and inter-glob cells are color selective? It would be useful to have summary statistics about narrowness of tuning, and shifts of tuning, that quantify the assertions in this statement here rather than later in the paper.

11. "The best model was similar regardless of the luminance of the stimulus set used to collect the neural data (Figure 6, left panels): for each stimulus set, the best model was one comprised of non-linear neurons that represent color space in a uniform fashion. Interglob cells were similarly best fit by a model comprising a uniform representation of color space (Figure 6, right panels)." Could this be an artifact of the discrete sampling of colors used for making the tuning curves?

12. "The interglobs are best described by the model consisting of broadly tuned neurons that are biased for the unique hues." This needs some explanation. What are the implications of this result?

13. The analysis in Fig 9 is weak. The sampling is not uniform across the circle, and the inference is particularly susceptible to non-uniformity in the metric used to sample colors. Unless there is some significant implication of this result, it can be left out.

14. The analysis in Fig 10 would probably be better captioned as Luminance Contrast Sensitivity. The issue of sensitivity to dark colors, such as brown, navy and maroon is too interesting to be given such short-shrift. The histogram comparison is fine because it is invariant to changes in tuning curves with luminance contrast, but it does not quantify how color sensitivity changes with luminance contrast. Also, in the absence of a proper analysis, the comment that "These results are consistent with the idea that the interglob population is sensitive to luminance contrast independent of the hue of the stimulus" is difficult to reconcile with the tuning curves of inter-glob cells in Fig 2. Clearly a closer analysis is required of how color tuning changes or not with luminance contrast.

15. There are two problems with the similarity analysis. First, I would need to see some justification that extended distances in CIELUV represent similarities between widely separated colors for humans or macaques. Second, I am not sure how to interpret the stress criteria. Ramsay, Takane, Young and others have developed maximum likelihood procedures for MDS that give probability estimates of fits etc, and it would be preferable to use these methods.

16. Decoding section. Given that the authors have just shown that glob cells can be selective for darker or lighter colors, and use a histogram analysis that enables them to ignore changes in color tuning with luminance contrast, I am not sure why different cells would not do decoding at different contrasts, so why look for decoding invariance across luminance contrast.

17. Figures 11-13. Given that the color signal from photoreceptors is 3-D, the authors need to identify under what scenario one would expect anything different from the results in Figure 11-13? Perhaps the authors should repeat the MDS etc exercise on the responses of the three (or four) classes of LGN cells, and see if there is any difference.

More minor comments:

The table detailing statistical comparisons is very helpful. Note however that the last two rows in this table state that p-value are Pending due to computation time. Perhaps this is a typo, because the tet states that p values are &amp;lg;0.0001 while referring to these two rows. Further, when reference to this table cannot be made as simple superscripts (a,b,c,d,e,f,g,h) -- it is not clear where to look for the corresponding footnotes. You should add Table 1, row a, etc.

The last analysis does raise some questions. For example, the difference in analyses underlying Figure 6 and Figure 7 was not clear. In the case of interglob regions, Figure 6 shows that more uniform pooling than Figure 7. Figure 6 does not show qualitative differences between pooling for globs and interglobs, but Figure 7 does.

Mutimedia:

We found the movies useful in seeing the 3D structure of the synthesized color space. However, would it perhaps be better, or possible, to provide a matlab/mathametica/python script that the readers could run to produce this figure. In this way, one can control the viewing angle and speed.
